# Size‐Dependent Pulmonary Impact of Thin Graphene Oxide Sheets in Mice: Toward Safe‐by‐Design

**DOI:** 10.1002/advs.201903200

**Published:** 2020-05-07

**Authors:** Artur Filipe Rodrigues, Leon Newman, Dhifaf Jasim, Sourav P. Mukherjee, Jun Wang, Isabella A. Vacchi, Cécilia Ménard‐Moyon, Alberto Bianco, Bengt Fadeel, Kostas Kostarelos, Cyrill Bussy

**Affiliations:** ^1^ Nanomedicine Lab Faculty of Biology, Medicine and Health University of Manchester Manchester Academic Health Science Centre Manchester M13 9PT UK; ^2^ National Graphene Institute University of Manchester Manchester M13 9PT UK; ^3^ Lydia Becker Institute of Immunology and Inflammation School of Health Sciences University of Manchester Manchester Academic Health Science Centre Manchester M13 9PT UK; ^4^ Nanosafety & Nanomedicine Laboratory Institute of Environmental Medicine Karolinska Institutet Stockholm 171 77 Sweden; ^5^ Science for Life Laboratory Department of Biochemistry and Biophysics Stockholm University Stockholm 171 65 Sweden; ^6^ University of Strasbourg CNRS Immunology, Immunopathology and Therapeutic Chemistry UPR 3572 Strasbourg 67 084 France; ^7^ Catalan Institute of Nanoscience and Nanotechnology (ICN2) Campus UAB Bellaterra Barcelona 08193 Spain

**Keywords:** graphene oxide, inflammation, lung, macrophages, mice, RNA sequencing

## Abstract

Safety assessment of graphene‐based materials (GBMs) including graphene oxide (GO) is essential for their safe use across many sectors of society. In particular, the link between specific material properties and biological effects needs to be further elucidated. Here, the effects of lateral dimensions of GO sheets in acute and chronic pulmonary responses after single intranasal instillation in mice are compared. Micrometer‐sized GO induces stronger pulmonary inflammation than nanometer‐sized GO, despite reduced translocation to the lungs. Genome‐wide RNA sequencing also reveals distinct size‐dependent effects of GO, in agreement with the histopathological results. Although large GO, but not the smallest GO, triggers the formation of granulomas that persists for up to 90 days, no pulmonary fibrosis is observed. These latter results can be partly explained by Raman imaging, which evidences the progressive biotransformation of GO into less graphitic structures. The findings demonstrate that lateral dimensions play a fundamental role in the pulmonary response to GO, and suggest that airborne exposure to micrometer‐sized GO should be avoided in the production plant or applications, where aerosolized dispersions are likely to occur. These results are important toward the implementation of a safer‐by‐design approach for GBM products and applications, for the benefit of workers and end‐users.

## Graphical abstract

Bulk transcriptomics in lungs of mice after pulmonary exposure to graphene oxide (GO) sheets of varying lateral dimensions reveals size‐dependent adverse effects and provides valuable information for safer‐by‐design production of GO sheet suspension. Analysis of histopathology, material fate and bioaccumulation, cell recruitment, and cytokine secretion confirms more adverse effects with micrometric GO than with nanometric GO.

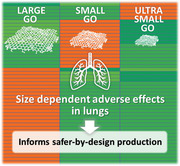

## 1 Introduction

Graphene and other two dimensional (2D) materials have generated substantial commercial interest due to their unique combination of physicochemical properties that are attractive for a wide range of applications.^[^ ^1^, ^2^ ^]^ Several methods of large‐scale production have been developed since the first isolation of graphene, yielding a range of different forms of graphene‐based materials (GBMs) whose properties such as lateral dimensions, thickness, and surface chemistry can vary significantly.^[^ ^3^, ^4^ ^]^ In particular, the low production cost of graphene oxide (GO) has contributed to the emergence of various commercial products based on GO‐based composites, dispersions, or spray coatings.^[^ ^5^ ^]^ As a result of this sustained commercial expansion, it is expected that exposure to GBMs via inhalation of aerosolized materials will increase, raising concerns about the potential impact of inhaled GBMs on human health.^[^ ^6^, ^7^ ^]^

In respect to the pulmonary route of exposure, graphene nanoplatelets (GNPs) have been the most widely studied GBMs to date, with several reports of transient inflammation after pulmonary exposure.^[^ ^8^, ^9^, ^10^, ^11^, ^12^, ^13^, ^14^ ^]^ In addition, Roberts et al. demonstrated that GNPs with large lateral dimensions (5–20 µm) have a more detrimental impact on the lungs than their smaller counterparts (<2 µm) following oropharyngeal aspiration,^[^ ^15^ ^]^ supporting the view that dimensions of these materials are a major determinant of their pulmonary toxicity. However, despite evidence that GNPs may persist in the lungs without being inflammogenic even after 6 weeks,^[^ ^16^ ^]^ longer term consequences of material persistence in the alveolar region, such as late onset disease or carcinogenesis, remain largely unknown.

In contrast, pulmonary responses to GO have not been studied to the same extent. Despite numerous in vitro studies on the toxicity of GO, there is not yet a consensus regarding the key properties underlying these effects due to the considerable variability in the physicochemical properties of the tested materials, as well as the use of different cell models.^[^ ^7^ ^]^ Indeed, while studies of the impact of GO on macrophage cell lines have suggested toxicity,^[^ ^17^ ^]^ we have recently reported that single to few‐layer GO is non‐cytotoxic for primary human macrophages (at the doses tested).^[^ ^18^ ^]^ On the other hand, endotoxin‐free GO sheets displayed cytotoxicity toward primary human neutrophils, resulting in the formation of so‐called extracellular traps.^[^ ^19^ ^]^ Furthermore, using a lung epithelial cell line, we demonstrated that large GO sheets (5–15 µm) elicited stronger cytotoxic responses compared to small GO sheets (50–200 nm).^[^ ^20^ ^]^ Similarly, Ma et al. reported enhanced inflammatory responses in the lungs of animals exposed to large GO sheets (750–1300 nm) when compared to small GO (50–350 nm).^[^ ^21^ ^]^ However, only the acute inflammatory response was assessed in the latter study, and questions regarding the potential long‐term effects of GO remain to be addressed.

With this in mind, the aim of the present study was to investigate whether lateral dimensions play a significant role in the pulmonary response to GO at short‐ and long‐term after single exposure in mice. Considering the literature, we hypothesized that micrometer‐sized GO sheets would induce a more deleterious pulmonary response than nanometer‐sized GO after single intranasal (i.n.) instillation (50 µg per mouse). To this end, we investigated both acute and chronic responses to three different GO materials produced with distinct lateral dimensions,^[^ ^22^ ^]^ for up to 90 days after single exposure. Pulmonary responses were characterized in terms of conventional histopathological markers, recruitment of immune cells, and secretion of inflammatory cytokines. Furthermore, we applied genome‐wide RNA sequencing (RNA‐seq)^[^ ^23^ ^]^ in order to evaluate potential differences between the three GO materials at the level of gene expression. However, the probability of inducing such effects could be reduced by a lower deposited dose of larger GO sheets in the alveolar region compared to smaller ones, as a result of greater clearance from the upper respiratory tract following intranasal administration. To test this possibility, we investigated the biodistribution and biodegradation of GO using a combination of inductively coupled plasma mass spectrometry (ICP‐MS) and Raman spectroscopy, and then contrasted the inflammatory effects of the three GO materials with their biokinetics (i.e., distribution, biotransformation, and/or clearance from the lungs). Under the tested conditions, we found that the largest GO sheets were causing the most adverse, widespread, and long‐term effects in mice, despite a lower lung deposition, but in agreement with persistent granulomas, while the pulmonary response to the smallest GO was predominantly acute and transient, in line with a faster clearance from the lungs.

## 2 Results

### 2.1 Characteristics of GO Sheets and Benchmark Materials

The three GO materials used in the present study were produced under endotoxin‐free conditions,^[^ ^22^ ^]^ and yielded single to few‐layer sheets with similar surface chemistry and thickness, but with varying lateral dimensions (full characterization was previously reported in ref. [22] indicative values in Table S1, Supporting Information). Large GO (l‐GO) was comprised of micrometer‐sized sheets ranging between 1 and 30 µm in lateral dimensions, whereas small GO (s‐GO) was made of sheets between 50 nm and up to 2 µm. The third materials, so‐called ultra‐small GO (us‐GO) sheets were all smaller than 300 nm, and no micrometer‐sized sheets were observed in these samples. Finally, multi‐walled carbon nanotubes (MWCNT; Mitsui‐7), classified by the International Agency for Research on Cancer (IARC) as a potential human carcinogen on the basis of numerous animal studies,^[^ ^24^, ^25^ ^]^ was included as a benchmark material, known to induce chronic inflammation and fibrosis. Notably, these nanotubes have lengths comparable to the lateral dimensions of the l‐GO tested here, ranging from 1 to 20 µm (median = 3.86 µm), and a high aspect ratio, with an average width of 49 ± 13.4 nm.^[^ ^24^ ^]^

### 2.2 Biodistribution of GO Sheets After Intranasal Instillation in Mice

**Figure 1:**
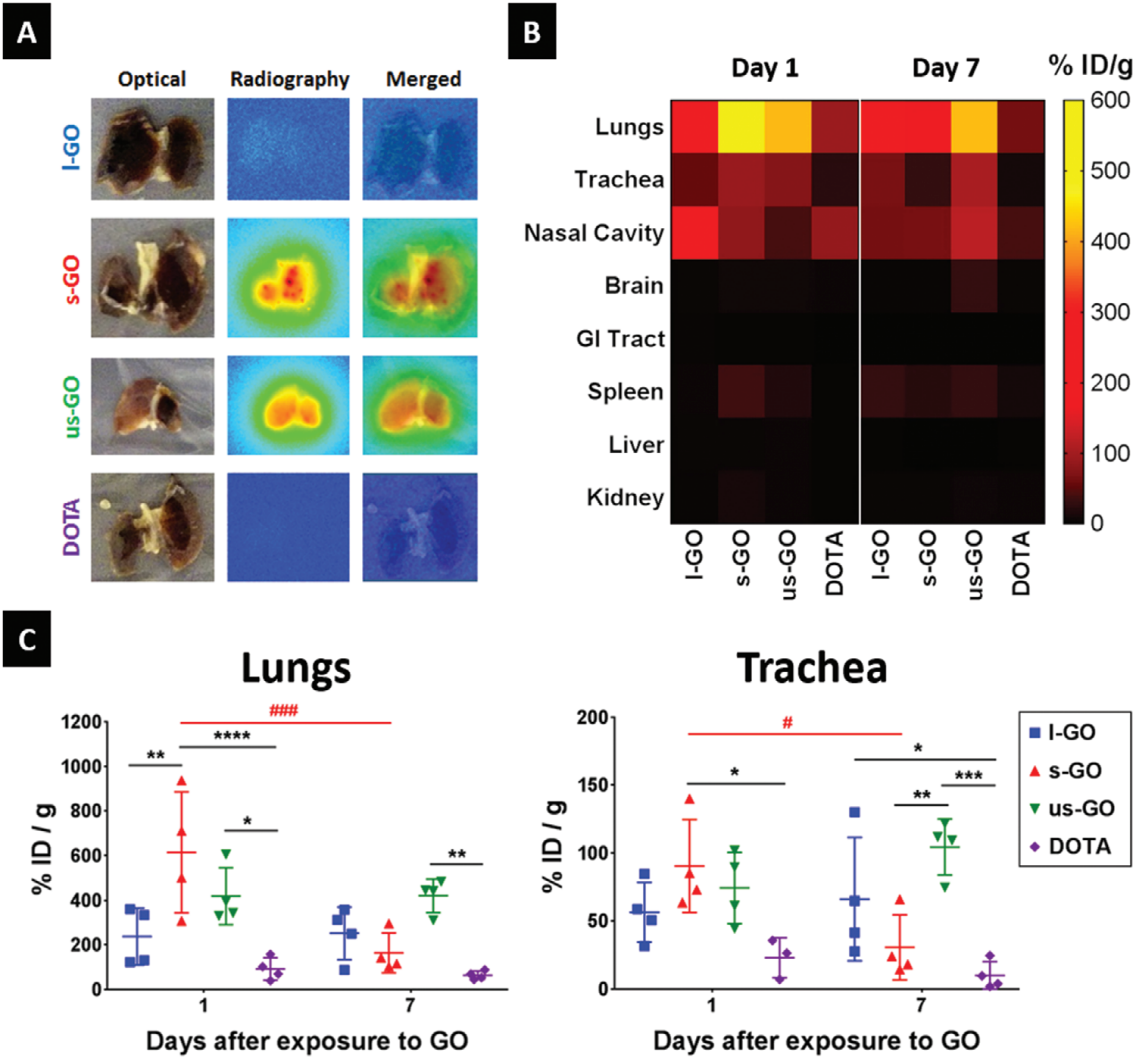
GO sheets translocate to the lower respiratory tract after intranasal instillation. Mice were instilled with DOTA‐functionalized GO sheets labeled with ^115^In (natural) or ^111^In (radioactive), and the organs were collected 1 and 7 days after exposure. A) Autoradiography of lungs dissected from mice instilled with GO‐DOTA[^111^In] 1 day after exposure. B) Heatmap illustrates tissue distribution and persistence of GO‐DOTA[^115^In] in the respiratory tract compared to DOTA[^115^In] control, at days 1 and 7 after i.n. instillation. Each block represents the mean amount of GO in the respective organ, quantified by ICP‐MS, which was normalized as % instilled dose (ID) per gram of dry tissue. C) Quantification of ^115^In by ICP‐MS in the lungs and trachea from mice exposed to GO‐DOTA[^115^In] or the DOTA[^115^In] control reveals size‐dependent distribution of GO in the respiratory tract. Individual data points corresponding to each animal are plotted alongside mean ± SD (*n* = 4). Data were analyzed using a two‐way ANOVA test with post hoc Sidak's multiple comparisons test. Significant differences between treatments are plotted with (*), whereas differences over time are plotted with (#). In both cases, statistical significance is reported as: (*), *p* < 0.05; (**), *p* < 0.01; (***), *p* < 0.001; (****), *p* < 0.0001. Remaining organs are plotted in Figure S2, Supporting Information.

In order to estimate the lung burden induced by GO after i.n. instillation (50 µg per mouse), we functionalized GO with NH_2_‐PEG_4_‐DOTA (GO‐DOTA) as previously reported^[^ ^26^ ^]^ and chelated the GO‐DOTA complexes with metal isotopes (^111^In or ^115^In). Isotope labelling purity and stability in physiological media were tested using ^111^In (Figure S1, Supporting Information), which demonstrated the suitability of these probes for biodistribution studies. Qualitative estimation of biodistribution using autoradiography of lungs exposed to 50 µg GO‐DOTA[^111^In] complexes revealed different intensities (**Figure** **1**A), suggesting a size‐dependent translocation to the lower respiratory tract 1 day after i.n. instillation. While l‐GO translocated less than the other two GO materials, s‐GO seemed to translocate more effectively to the lungs compared to us‐GO. For quantitative biodistribution (up to 7 days after instillation), we use ICP‐MS after chelating the natural isotope ^115^In to the GO‐DOTA complexes (Figure 1B). We again observed a size‐dependent deposition in the lower respiratory tract (reported in the panel as lungs and trachea; Figure 1C), with l‐GO corresponding to the lowest deposited dose (7.01% ± 4.42%) and s‐GO sheets to the highest (17.48% ± 7.44%). At an intermediate level, 12.08% (± 5.77%) of instilled us‐GO sheets reached the lower respiratory tract (Table S2, Supporting Information), and persisted in both lungs and trachea up to the latest time point tested here (7 days post exposure). After normalizing by the lung dry weight, the uptake of GO sheets by the lungs was calculated to be 11.88 ± 6.38 mg g^−1^ for l‐GO, 30.72 ± 13.55 mg g^−1^ for s‐GO, and 20.92 ± 6.37 mg g^−1^ for us‐GO (Table S3, Supporting Information). Lung uptake of s‐GO and us‐GO was significantly greater than that of the DOTA control (*p* < 0.0001 for s‐GO and *p* = 0.0102 for us‐GO), which totaled 4.63 ± 2.52 mg g^−1^. Although the delivered dose to the lungs and trachea was lower than s‐GO and us‐GO, l‐GO was found to be persistent in the trachea, with a detected dose that was about 6.6 times greater than DOTA at day 7 post instillation (*p* = 0.0450). Similar retention was also observed for us‐GO, with detected doses in the lungs and trachea that were 21 (*p* = 0.0045) and 5.2 times (*p* = 0.0003) greater than DOTA, respectively. On the other hand, the amount of s‐GO in the lungs and trachea decreased by 73% (*p* = 0.0002) and 66% (*p* = 0.0191), respectively, between day 1 and 7, suggesting a greater clearance.

The lower amount of l‐GO found in the lower respiratory tract correlated with its greater detection in extra‐pulmonary organs such as the nasal cavity (*p* < 0.0037 compared to all other treatments) or the gastrointestinal (GI) tract (*p* = 0.0008 versus DOTA) at day 1 post exposure (Figure S2, Supporting Information). Significant translocation to the gastrointestinal tract was also observed for s‐GO (*p* = 0.0427) compared to DOTA. Moreover, the amount of s‐GO in the kidney was 4.9 and 3.6 times greater than DOTA (*p* = 0.0159) and l‐GO (*p* = 0.0315), respectively. These results suggested the translocation of s‐GO to the bloodstream, with subsequent urinary excretion following glomerular filtration, in line with previous observations following other administration routes.^[^ ^26^, ^27^ ^]^ However, this translocation was not accompanied by a significant retention in organs of the reticuloendothelial system such as liver or spleen, compared to the DOTA control (*p* = 0.5014). The continuing decrease in signal from the nasal cavity after l‐GO exposure suggested that these materials were efficiently eliminated via mucociliary clearance. On the other hand, us‐GO translocated significantly from the nasal cavity to the brain over time (*p* = 0.0005), with a retained dose at day 7 post instillation that was at least 8 times higher than the other treatments (*p* < 0.0002), most likely because of the smaller dimensions of us‐GO. No significant accumulation in the remaining extra‐pulmonary organs was observed for any material compared to the DOTA control. These results suggested that lateral dimensions affect the pulmonary deposition of GO sheets and their biokinetics, which may ultimately determine their biological impact. Because the amount of GO reaching the lungs was randomly distributed in each lobe (Figure S2, Supporting Information), we decided for each animal to sample each lobe and then pool these samples in order to further characterize the overall pulmonary response (see details below).

### 2.3 Tissue Response to GO Sheets after Intranasal Instillation

**Figure 2:**
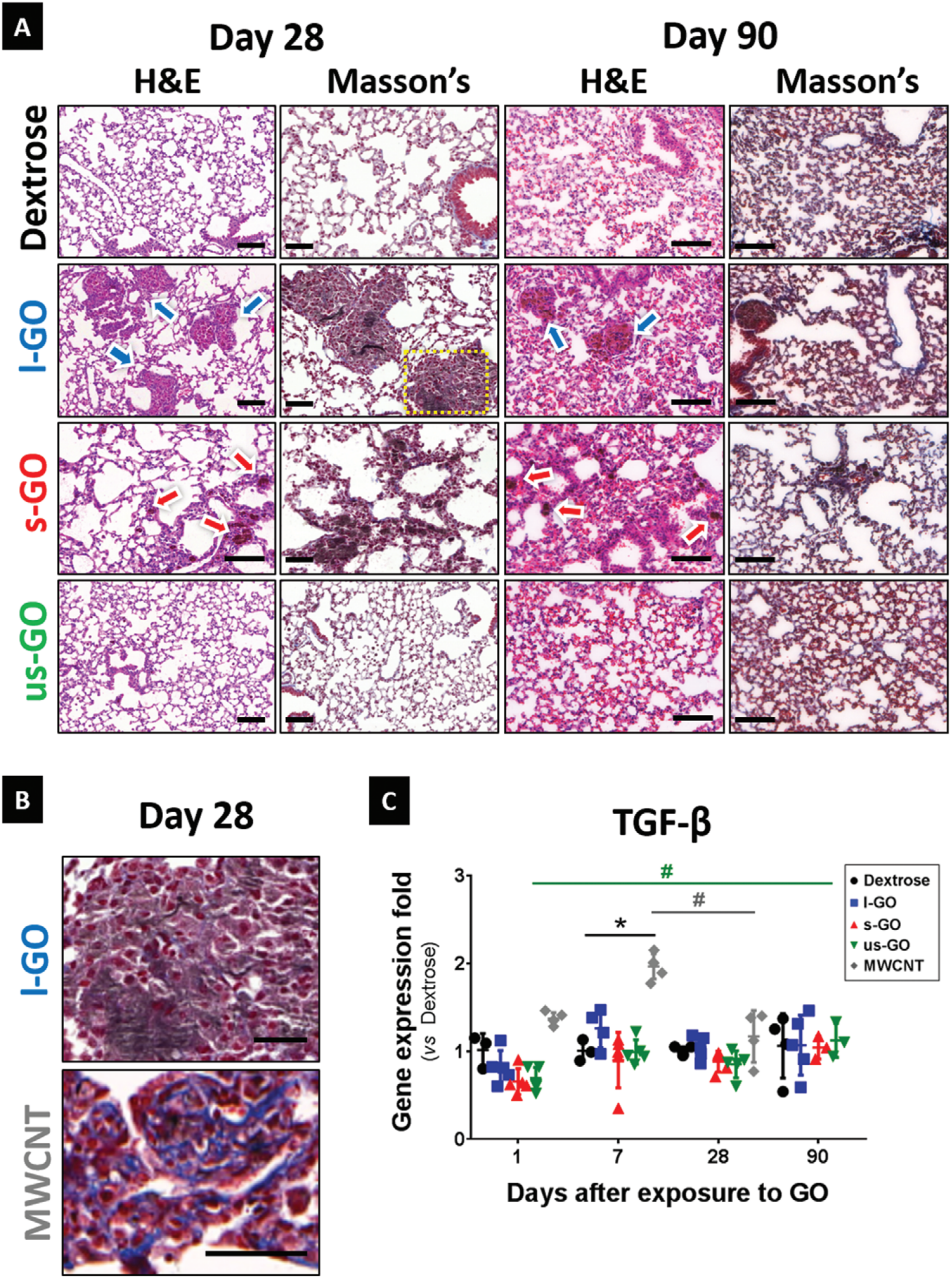
Pulmonary exposure to GO induces size‐dependent granulomatous inflammation in the lungs. Lung sections from mice instilled with GO were extracted at 1, 7, 28, and 90 days after i.n. instillation. A) Representative images of sections stained with H&E and Masson's trichrome at days 28 and 90 were acquired using a 20x magnification. Remaining time points are shown in Figure S4, Supporting Information. Arrows indicate areas of significant immune cell infiltration in response to the presence of GO, with alveolar wall thickening and granuloma formation. Scale bars = 100 µm. B) Despite granuloma formation, l‐GO failed to induce significant collagen deposition, a typically hallmark for pulmonary fibrosis. Images correspond to areas highlighted with a yellow box in the l‐GO group (MWCNT group is presented in Figure S4, Supporting Information). Scale bars = 50 µm. C) Small portions of each individual lobe were dissected for RNA extraction and RT‐qPCR. Gene expression of TGF‐*β* was quantified as relative fold increase compared to the negative control (Dextrose) at each time point. Data are presented as individual points corresponding to each animal, alongside mean values ± SD are presented (*n* = 4–5). Statistical analysis was performed using a Kruskal–Wallis test with post hoc Dunn's multiple comparisons test against the negative control: (*), *p* < 0.05. The temporal variation of TGF‐*β* expression in the lungs was also analyzed using a Kruskal–Wallis test with post hoc Dunn's multiple comparisons test: (#), *p* < 0.05.

Pulmonary responses to GO sheets were assessed at days 1, 7, 28, and 90 after exposure (**Figure** **2**; Figure S3, Supporting Information). Lung histopathology indicated an acute inflammatory response at days 1 and 7 post instillation, which was characterized by alveolar wall thickening with infiltration of predominantly mononuclear cells in the interstitium for all three GO materials. Chronic inflammation was evidenced by the development of non‐necrotizing peri‐bronchiolar granulomas in the lungs of mice exposed to s‐GO and l‐GO at days 28 and 90 post exposure (Figure 2A). These granulomas were localized only in areas with significant agglomeration of GO. Moreover, the size of these granulomas correlated with the lateral dimensions of the administered materials (the larger the GO sheets, the bigger the granulomas). Quantification of areas showing interstitial infiltration revealed a size‐dependent effect of GO materials, with l‐GO inducing the most significant pulmonary infiltration at days 28 (*p* = 0.0218) and 90 (*p* = 0.0002) compared to the negative control (Figure S3, Supporting Information). Similarly, s‐GO also triggered a significant granulomatous response 28 days after instillation (*p* = 0.0203). However, this was followed by a reduction of the infiltrated areas after 90 days (*p* = 0.0187). In contrast, us‐GO did not induce a significant granulomatous response in the lungs, with the parenchyma recovering completely from acute interstitial inflammation at day 28 post instillation, and showing similar histological features to mice treated with the negative (vehicle) control. As expected on the basis of previous reports, MWCNTs (MWCNT‐7), used here as positive control, induced both acute and chronic interstitial infiltration (Figure S3, Supporting Information), showing inflammatory patterns similar to or worse than l‐GO at 28 days after exposure (*p* < 0.0293). Noticeably, chronic persistent inflammation in response to MWCNTs only occurred in areas of material agglomeration in a similar fashion as for l‐GO and s‐GO.

Because chronic histopathological inflammation coincided with the apparent persistence of GO, as evidenced by the presence of dark brown matter at 90 days post exposure, we also assessed whether lung fibrosis was associated to granulomas at the two latest time points. To this end, Masson's trichrome staining was used to reveal the deposition of collagen (Figure 2B). As expected, MWCNTs promoted interstitial fibrosis in the alveolar region, already by day 7 (Figure S3, Supporting Information), which coincided with areas of granulomatous inflammation. In agreement with this data, exposure to MWCNTs also increased the expression of the known fibrosis marker TGF‐*β* by 96% (*p* = 0.0453) compared to the vehicle control (Figure 2C). On the other hand, none of the three GO materials induced any obvious collagen deposition or upregulation of TGF‐*β* compared to control, in spite of the formation of persistent granulomas after exposure to l‐GO or s‐GO.

### 2.4 Evidence of Biotransformation of GO Sheets in the Lungs

**Figure 3:**
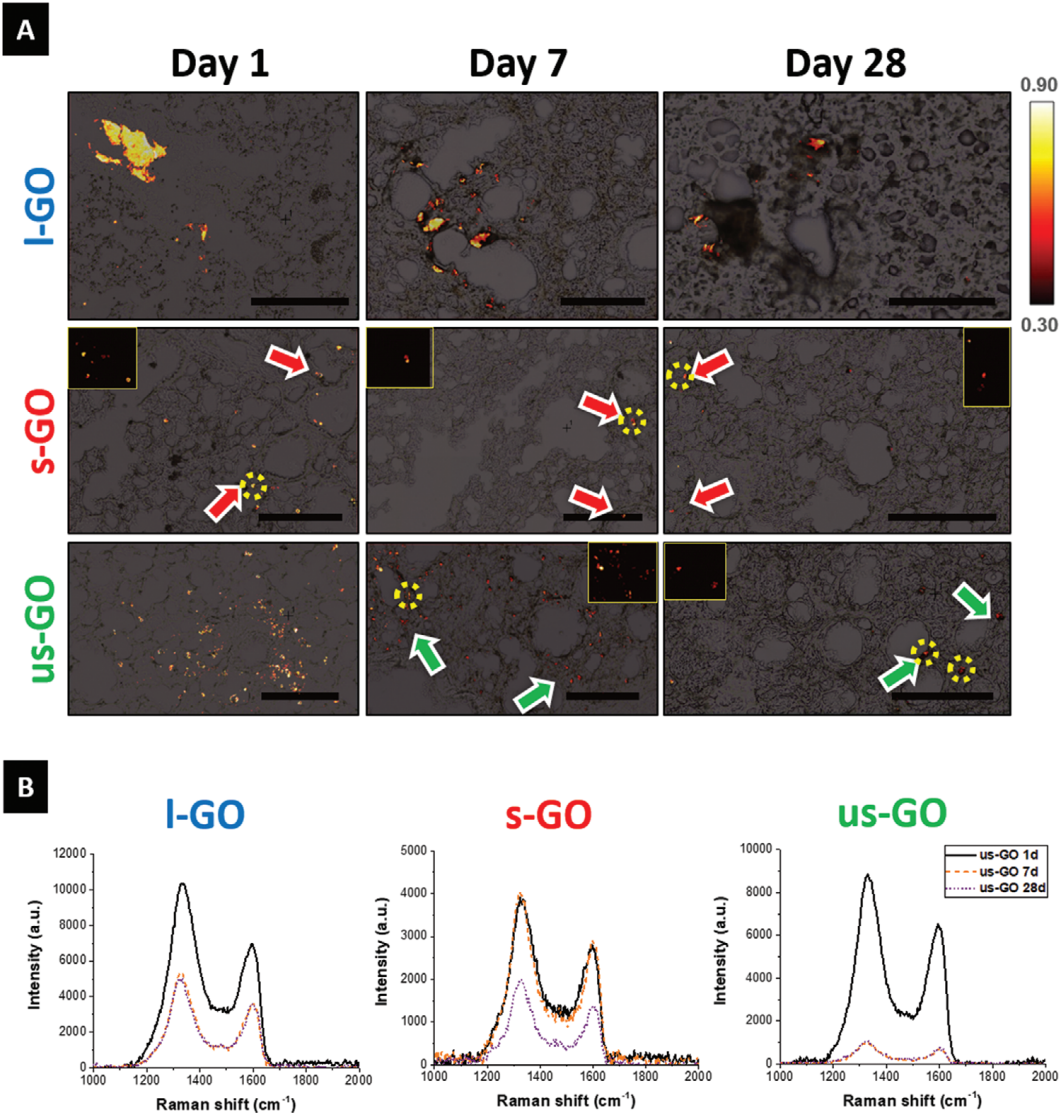
GO sheets undergo biodegradation and/or clearance from the lungs after pulmonary exposure. Lung cryosections were obtained from mice exposed to l‐GO, s‐GO, and us‐GO. A) Raman maps are plotted overlaying bright‐field images, with the intensity scale referring to the correlation between the acquired spectrum for each pixel of the map and the reference spectrum of GO presented in Figure S4, Supporting Information. Presence of s‐GO and us‐GO is emphasized in inset figures, corresponding to areas highlighted in yellow. Scale bars = 100 µm. B) Representative Raman spectra corresponding to the average spectrum at each time point evidence biotransformation of GO in the lungs, with decreased signal intensity and spectroscopic changes including the shifted position of the G band and the variation in the relative intensity of the D band.

Lack of fibrosis despite the persistence of GO materials in the lungs could be attributed to the ability of the host tissue to transform the foreign material into a less reactive material.^[^ ^28^ ^]^ To evaluate this possibility, we performed Raman mapping of lung sections from exposed animals (**Figure** **3**). The presence of GO in the lungs produced a specific Raman signature (Figure S4, Supporting Information), whose evolution over time can be used as an indicator of biotransformation resulting from the loss of the crystalline structure of the material. We therefore analyzed the Raman “fingerprints” of GO in the lungs by Raman spectroscopy‐based imaging (Figure 3). At day 1, whilst l‐GO was predominantly identified as large agglomerates associated with the lung parenchyma, s‐GO and us‐GO were found in the airways, mainly within circular features resembling individual cells (Figure 3A). In agreement with the results obtained from ICP‐MS (Figure 1C) and autoradiography (Figure 1A) experiments, a more widespread and persistent (up to 7 days) signal could be observed over time for us‐GO compared to s‐GO, suggesting a slower clearance of the former compared to the latter material. Interestingly, all three GO materials could still be detected up to 28 days after administration, though the Raman signal corresponding to the starting materials was dampened from day 1 to 28, suggesting that the structure of the materials was different from the starting materials. Besides the indication of clearance, the decreased Raman signal intensities over time suggested that all three GO materials underwent biotransformation^[^ ^29^ ^]^ (Figure 3B; Figures S5–S7, Supporting Information). Fine spectral analysis revealed that all GO materials exhibited time dependent spectroscopic changes. Despite the apparent increase from 1.32 ± 0.09 to 1.38 ± 0.18, us‐GO did not display significant changes in level of crystalline disorder, revealed by the ratio between the D and G bands (*I*_D_/*I*_G_).^[^ ^30^ ^]^ Nevertheless, this material suffered the most dramatic reduction in Raman signal intensity, which suggested that either us‐GO sheets were cleared from the lungs or biotransformation was leading to their disappearance via biodegradation. Additional spectroscopic features indicative of biotransformation were noted, such as the dislocation of the G band from 1594 to 1603 cm^−1^ at day 28 post exposure (*p* = 0.0211) suggesting the emergence of a D’ band that is merged to the G band. On the other hand, s‐GO was the least affected material since no spectroscopic features changed significantly over time, including a constant *I*_D_/*I*_G_ ratio at 1.35 ± 0.12 after 28 days. Interestingly, l‐GO revealed the most significant spectroscopic changes, as evidenced by Raman analysis, including a decrease in *I*_D_/*I*_G_ band from 1.48 ± 0.07 to 1.38 ± 0.08 at day 28 post instillation (*p* = 0.0332) and the dislocation of the G band from 1594 to 1602 cm^−1^ (*p* = 0.0181). This material presented a higher *I*_D_/*I*_G_ band at day 1 post exposure compared to s‐GO and us‐GO, despite their similar structure before administration.^[^ ^22^ ^]^ Following this initial increase, a decreased *I*_D_/*I*_G_ ratio at day 28 suggested that l‐GO had become more amorphous than the other two materials, with defects becoming more widespread with time, thus reducing the sp^2^ conjugated areas (i.e., loss of crystalline structure).^[^ ^30^ ^]^

### 2.5 Inflammatory Response to GO Sheets in the Airways and Lungs

Intranasal instillation with GO dispersions (50 µg per mouse) did not result in acute toxicity, although mice instilled with s‐GO and us‐GO suffered mild weight loss, in line with higher lung burden, followed by full recovery within 4 days post exposure (Figure S8A, Supporting Information). Acute pulmonary responses were assessed by measuring total protein and lactate dehydrogenase (LDH) release to the airways. The analysis of bronchoalveolar lavage (BAL) fluid revealed size‐dependent disruption of the air‐blood barrier, as suggested by the release of proteins to the airways (Figure S8B, Supporting Information). Nevertheless, no statistical significance was observed compared to the negative control, and only MWCNTs inducing 2.7 times more protein exudation 7 days after instillation (*p* = 0.0325). Exposure to any of the three GO materials was followed by rapid recovery after 7 days, which was maintained for up to 28 days. Cytotoxicity levels of s‐GO at 7 days after administration were 2.4‐fold greater than vehicle control (*p* = 0.0034), suggesting that the biological response to GO may develop over time (Figure S8C, Supporting Information), in a similar fashion to the observed granuloma formation (Figure 2). MWCNTs did not induce significant acute effects, despite a mild increase in cytotoxicity 1 day after administration (*p* = 0.0511).

Acute responses were further evaluated by quantifying the recruitment of leukocytes in the airways by differential staining of the cells recovered from bronchoalveolar lavage at days 1, 7, and 28 post exposure (Figure S9, Supporting Information). All three GO materials induced significant recruitment of polymorphonuclear cells (PMN), with s‐GO eliciting the most acute effects compared to the vehicle control at day 1 (*p* = 0.0025), in line with a higher amount of translocated materials to the lungs (Figure 1C). At day 7, l‐GO promoted significant infiltration of PMNs (*p* = 0.0313), similarly to MWCNTs (*p* = 0.0063), thus suggesting that the typical inflammatory response to foreign materials could be prolonged. Nevertheless, PMN infiltration to the airways was no longer detected at 28 days after exposure, which suggested recovery from the inflammatory stimulus. These results are in contrast with the histopathological evidence of granuloma formation (discussed above). A possible explanation could reside in a reduction in the number of inflammatory cells that are recoverable by lavage versus those cells that are recruited to the lungs.

**Figure 4:**
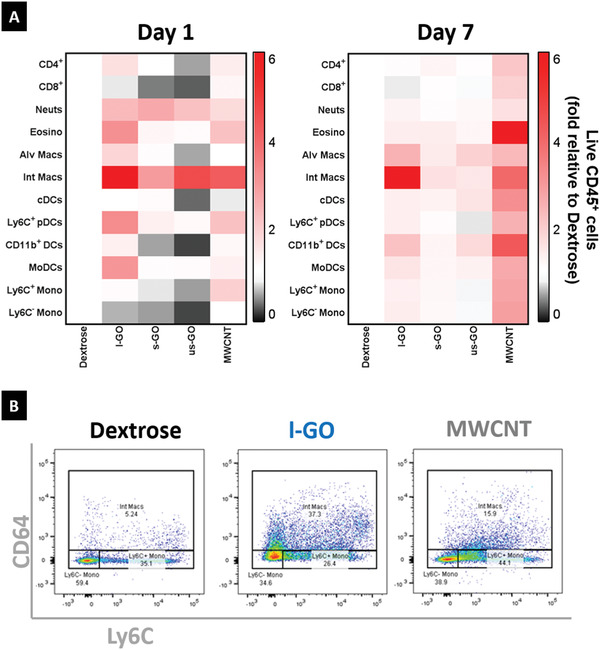
GO sheets trigger acute recruitment of interstitial macrophages after pulmonary exposure. Lungs of mice exposed to carbon nanomaterials were dissected 1 and 7 days after i.n. instillation, and digested in order to extract cells for flow cytometry. A) Heatmap showing the variation in abundance of immune cell populations relatively to the negative control (Dextrose). Each block represents the mean value, after normalization to the average total number of cells in a specific immune cell population (*n* = 4). Absolute cell counts for each immune cell population corresponded to the product of the relative abundance in percentage of live CD45^+^ cells and the total number of live cells determined by Trypan Blue assay. B) Representative images showing the relative abundance of myeloid cells (CD11b^+^CD11c^−^) 1 day after exposure to carbon nanomaterials (see Figure S10, Supporting Information for gating strategy). Exposure to l‐GO and MWCNT increased the relative amount of interstitial macrophages (CD64^+^), in contrast to a decreased population of patrolling monocytes (CD64^−^Ly6C^−^), thus evidencing an acute inflammatory response.

To fully characterize the inflammatory response to GO sheets in the lungs, we quantified the recruitment of immune cells to the lung parenchyma using flow cytometry (Figure S10, Supporting Information). Exposure to any of the three GO materials resulted in a general increase in CD64^+^CD11b^+^ interstitial macrophages and neutrophils (**Figure** **4**A), although only l‐GO triggered significant recruitment of interstitial macrophages (*p* = 0.0025), monocyte‐derived dendritic cells (moDCs, *p* = 0.0213), and activated plasmacytoid DCs (*p* = 0.0163) 1 day after exposure (Figure S11, Supporting Information). Nevertheless, immune cell recruitment was mostly resolved 7 days after instillation of s‐GO and us‐GO, whereas l‐GO seemed to promote a prolonged infiltration of monocyte‐derived CD11b^+^ cells (Figure S11, Supporting Information), suggesting the establishment of a more chronic inflammatory response to this type of GO. In contrast, MWCNTs were not as efficient in stimulating granulomatous interstitial infiltration as l‐GO (Figure 4B), despite the apparent increase in interstitial macrophages (*p* = 0.5884). Similarly to the cytokine expression profile (see below), mice exposed to MWCNTs did not induce significant changes in immune cell recruitment, despite a general increase at 7 days post exposure that suggested a chronic inflammation characterized by an increased population of eosinophils (*p* = 0.0901).

**Figure 5:**
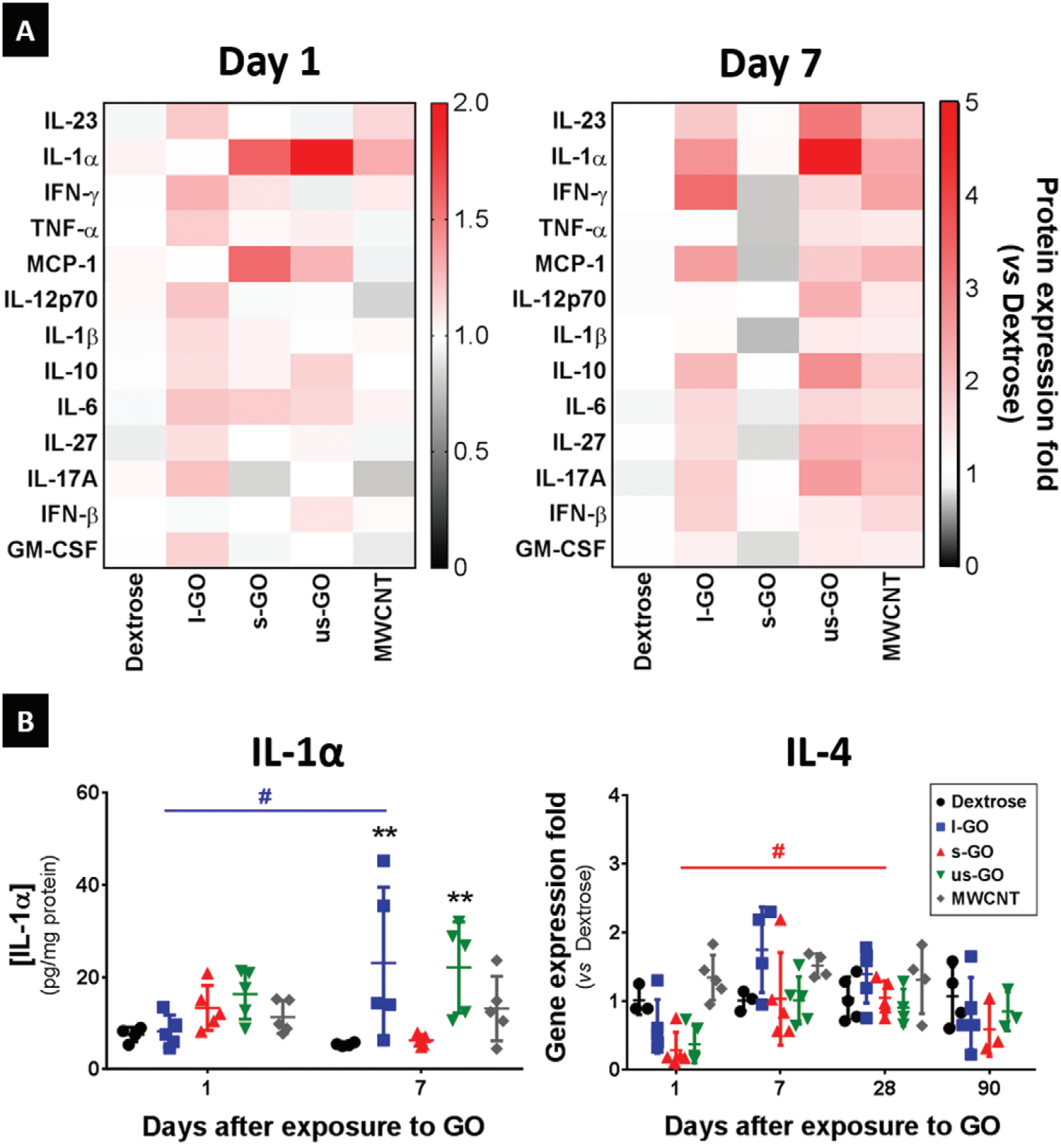
Inflammatory responses to l‐GO in the lungs are characterized by the secretion of Th1 cytokines. Small portions of each individual lobe were dissected for protein and RNA extraction. A) Heatmap showing the cytokine and chemokine expression levels of each treatment, normalized by protein content. Each block corresponds to the median value, after normalization to the average protein expression level in the negative control (Dextrose). Cytokine and chemokine expression levels were determined using a LEGENDplex Mouse Inflammation Panel kit, and normalized to protein concentration determined by BCA assay. B) Individual data points corresponding to each animal are plotted alongside mean values ± SD (*n* = 4–5). Statistical analysis of data acquired using the LEGENDplex assay was performed using a 2‐way ANOVA test with post hoc Sidak's multiple comparisons test, whereas gene expression of IL‐4 was analyzed using a Kruskal–Wallis test with post hoc Dunn's multiple comparisons test. In both cases, each treatment was only compared against the negative control: (*), *p* < 0.05; (**), *p* < 0.01. The temporal variation in the expression of IL‐4 was also analyzed using a Kruskal–Wallis test with post hoc Dunn's multiple comparisons test: (#), *p* < 0.05.

Cytokine expression, measured using a multiplex array, revealed different pro‐inflammatory responses for the different materials at 1 and 7 days after i.n. instillation (**Figure** **5**). All three GO materials induced only a mild acute inflammatory response, with more pronounced effects observed at day 7 after exposure. Interestingly, and in agreement with histopathological observations, most of the measured cytokines were upregulated by exposure to l‐GO, with the most dramatic effects occurring after 7 days. This inflammatory response was characterized by a significantly increased expression of IL‐1*α* (*p* = 0.0032), IL‐6 (*p* = 0.0406), IL‐23 (*p* = 0.0377), IFN‐*β* (*p* = 0.0398), and IFN‐*γ* (*p* = 0.0117), compared to mice treated with 5% dextrose, 7 days after instillation (Figure S12, Supporting Information). Exposure to s‐GO did not result in significant changes in cytokine expression, despite the apparent increase of IL‐1*α* (*p* = 0.6654) and the chemokine MCP‐1 (*p* = 0.3053), 1 day after administration (Figure 5). On the other hand, us‐GO elicited a mild inflammatory response, stimulating the expression of IL‐1*α* to similar levels to those observed in mice exposed to l‐GO, which represented a 3.1‐fold increase relative to vehicle control at day 7 post instillation (*p* = 0.0057). The latter cytokine seemed to be upregulated in a size‐dependent manner at early time‐points, with us‐GO inducing the greatest increase at 1 day after instillation albeit without statistical significance (*p* = 0.2816). An opposite trend was found regarding the expression of IL‐4, with all GO materials inducing a decreased expression at day 1 despite the non‐significance compared to vehicle control (*p* = 0.1244). This inhibition was followed by a significant increase in IL‐4 expression in mice exposed to s‐GO (*p* = 0.0265), to levels similar to the vehicle control at day 28 post exposure. Finally, MWCNTs did not induce statistically significant changes in cytokine production, despite an apparent increase of pro‐inflammatory cytokines at day 7 post exposure.

### 2.6 Transcriptomics Analysis of Lungs Exposed to GO Sheets

To gain further molecular insight into the pulmonary responses to the three different GO materials, we performed RNA sequencing on total RNA extracted from lung tissues at days 1, 7, and 28 post exposure. Sequencing was performed using the Illumina HiSeq2500 platform, and sequencing reads were aligned against the *Mus musculus* (GRCm38) genome. RNA sequencing generated 500 GB of raw sequence data with very high sequencing depth (>15 million reads per sample). Differentially expressed genes (DEGs) displaying a fold change (logFC) ≥ 0.5, and *p* ≤ 0.05 were considered for functional (bioinformatics) analysis. Enriched networks, molecular functions, and pathways were elaborated using the Ingenuity Pathway Analysis (IPA) tool.^[^ ^31^ ^]^ Venn diagrams were used to group unique and common DEGs identified in mice exposed to GO materials at 1, 7, and 28 days post exposure (Figure S13A, Supporting Information). RNA sequencing results displayed in this manner revealed distinct time‐dependent patterns of gene expression for all three GO materials. Markedly, gene expression changes were attenuated at 28 days post exposure for us‐GO when compared to days 1 and 7, in line with the observation that histopathological signs of inflammation were largely resolved at this time‐point. In contrast, for l‐GO, the number of DEGs (both up‐ and downregulated genes) continued to increase up to 28 days post exposure, indicative of a long‐lasting pulmonary response to this material.

**Figure 6:**
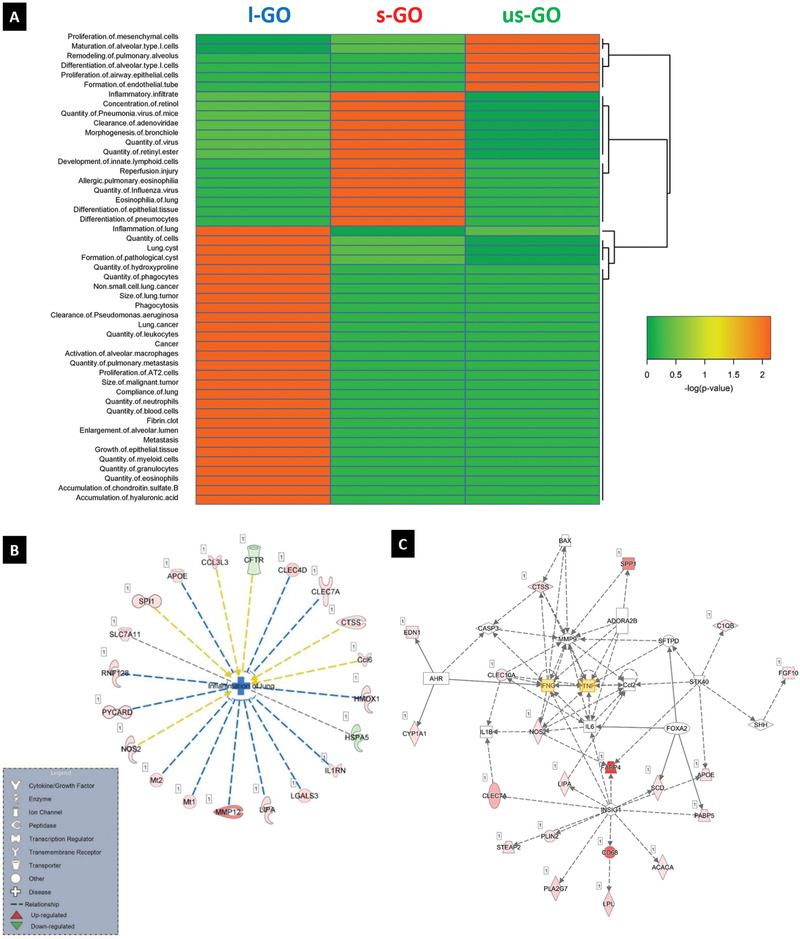
RNA‐seq analysis reveals size‐dependent effects of GO in inflammation‐related pathways. A) Hierarchical clustering analysis of the top diseases and biofunctions pathways identified by IPA in lung tissue samples of mice exposed for 28 days to us‐GO, s‐GO, and l‐GO. DEGs having ≥0.5 log fold change and ≥ 0.05 FDR were included in the analysis. The color coding in the heatmap depicts the *p*‐values for the pathways shown. B) IPA analysis was conducted on DEGs identified at day 28 post exposure. The pathway inflammation of the lung was significantly affected in lungs of animals exposed to l‐GO. Red denotes upregulated genes, and green denotes genes that are downregulated. C) Network analysis of DEGs identified in the lungs of mice at 28 days post‐exposure to l‐GO. IPA suggested that IFN‐*γ* and TNF‐*α* (highlighted in yellow) are main regulators of the affected pathways by l‐GO.

In order to compare the impact of the three different GO materials, we then performed hierarchical clustering analysis of the top “diseases and biofunction” pathways identified by IPA at day 28 (**Figure** **6**A). The data showed remarkable lateral dimension‐dependent differences in affected pathways. Notably, while all three GO materials affected pathways related to inflammation or immune cell function, several pathways annotated as “lung cancer related” in IPA were only affected in mice exposed to l‐GO for this time point (i.e., not found after either us‐GO or s‐GO exposure). Further analysis of the latter pathways revealed genes encoding matrix metalloproteinases (such as MMP3) and cathepsins (including CTSB and CTSZ), as well as the gene encoding the protein serpin B2 (also known as plasminogen activator inhibitor type 2 or PAI‐2). Similarly, when the top most‐affected “canonical pathways” were clustered, distinct size‐dependent differences were observed at day 28 post exposure (Figure S13B, Supporting Information). The top “diseases and biofunction” pathways identified by IPA at day 1, 7, and 28 for each individual GO material are reported in Figures S14–S16, Supporting Information. Numerous pathways related to lung inflammation were affected, which agreed well with other experimental data reported above.

To illustrate these findings, the genes in the “inflammation of the lung” pathway that were found to be significantly perturbed by l‐GO at day 28 are depicted in Figure 6B. These include genes such as PYCARD, encoding the protein ASC (apoptosis‐associated speck‐like protein containing a caspase recruitment domain) a key adaptor of the inflammasome^[^ ^32^ ^]^; the gene encoding the IL‐1 receptor antagonist; genes encoding members of the C‐type lectin/C‐type lectin‐like domain (CTL/CTLD) superfamily^[^ ^33^ ^]^; or the gene encoding MMP12 (Matrix Metallopeptidase 12, also known as macrophage metalloelastase or macrophage elastase).^[^ ^34^ ^]^ Notably and in agreement with the histological data, none of the GO materials were found to affect fibrosis pathways. Finally, network analysis of DEGs performed at day 28 post l‐GO exposure revealed that both TNF‐*α* and IFN‐ɣ acted as central nodes or putative regulators of the affected genes, as shown in Figure 6C; Figures S14–S16, Supporting Information.

## 3 Discussion

Understanding the potential hazard associated to a pulmonary exposure to GBMs or other 2D materials is a major goal of scientists, regulatory agencies, and industrial stakeholders. However, while there are several studies on the pulmonary impact of GNPs, very few studies have been performed to date on other GBMs including GO,^[^ ^7^ ^]^ and they focused primarily on acute response with a maximum follow‐up of 14–21 days post exposure.^[^ ^35^, ^36^ ^]^ Herein we studied the biodistribution, biotransformation, and pulmonary response to GO in mice for up to 90 days after single i.n. instillation of GO sheets with varying lateral dimensions (50 µg per animal). We demonstrated a size‐dependent translocation to the lower airways. In the airways, the formation of material agglomerates was also size‐dependent: the larger the materials, the greater the agglomeration. However, regardless of their primary lateral dimensions, all materials were submitted to clearance and had undergone a time‐dependent biotransformation, which left behind materials with an altered crystalline structure. In terms of pulmonary response, adverse outcomes were also size‐dependent; with micrometer‐sized GO inducing more pronounced and long‐lasting effects when compared to nanometer‐sized GO. Importantly, only the material suspensions containing micrometer‐sized GO sheets triggered the formation of granulomas that persisted for up to 90 days. Yet no significant pulmonary fibrosis was observed after exposure to these large materials.

### 3.1 Lateral Dimensions Govern the Biodistribution and Pulmonary Responses to GO

In comparison to s‐GO or us‐GO, greater amounts of l‐GO were detected in the nasal cavity and GI tract, while lower amounts were measured in the lungs. These findings suggest that micrometer‐sized GO sheets were less able to penetrate the lung airways than the other two materials, and more likely to undergo mucociliary clearance from the upper airways, as previously postulated when considering solely the nanomaterial size.^[^ ^37^ ^]^ However, despite the lowest dose delivered to the lower airways, l‐GO induced the strongest granulomatous inflammation (histopathology) and the highest impact at the molecular level (whole lung RNA sequencing), hence suggesting a higher material reactivity, in agreement with previous studies reporting larger materials to be more deleterious.^[^ ^15^, ^20^, ^21^, ^38^ ^]^ In addition, granuloma formation after l‐GO exposure appeared to be strongly correlated with the formation of material agglomerates, which were the largest amongst the three materials and persisted for up to 90 days, as revealed by Raman imaging.

The greatest lung burden was found in s‐GO exposed mice, which in turn elicited the strongest response in the airways. Indeed, a greater neutrophil infiltration to the airways correlated well with a greater delivered dose; however, s‐GO did not exert a strong inflammatory cytokine secretion or molecular impact in lungs, suggesting that the biological effects of s‐GO might reside primarily in the airways rather than the lung parenchyma. As in l‐GO exposed mice, material agglomerates persisting for up to 90 days were found in s‐GO exposed animals. Comparable material agglomerates persisting over 90 days were also reported by Bengtson et al. after single i.t. instillation.^[^ ^39^ ^]^ Likewise, Li et al. have demonstrated a similar persistence of small GO sheets (10–800 nm) for an equivalent period of time (3 months), also after i.t. instillation.^[^ ^40^ ^]^ In that study, GO sheets were causing acute lung injury with dose‐dependent inflammation and cytotoxicity, followed by pulmonary fibrosis starting 1 month after exposure. Despite having similar lateral dimensions, the s‐GO sheets used here exerted a much milder response (i.e., acute inflammation without fibrosis), which could be explained by a 3.5 times lower delivered dose to the lungs than in Li et al.^[^ ^40^ ^]^

Despite the presence of materials in the lower airways evidenced by Raman imaging at 1 and 7 days, us‐GO was the material that least interacted with the lung epithelium, supporting the idea that material agglomeration and persistence were size‐dependent. Differences in lung burden between us‐GO and s‐GO (or l‐GO) can be first explained by the greater ability of us‐GO to translocate to extra‐pulmonary organs (such as the brain, possibly via the olfactory tract^[^ ^37^ ^]^), as measured by ICPMS. Secondly, us‐GO sheets were distributed more evenly within the lung airways, rarely forming material agglomerates, as revealed by Raman imaging. This pattern would favor us‐GO likelihood of being taken up by lung macrophages and drained to lymph nodes.^[^ ^37^ ^]^ Early material presence (i.e., day 1 and 7) could hence be due to a slow uptake by airway macrophages, with degradation and/or clearance from the alveolar region occurring over several months, as suggested for other nanomaterials.^[^ ^41^ ^]^ In fact, Raman signal intensity for us‐GO in lung sections was reduced with time. Overall, the fate (i.e., biodistribution, agglomeration and clearance profile) of us‐GO sheets in the lungs agreed well with the observed biological response to these materials (i.e., mild and transient inflammation, with full histological recovery by day 28 after exposure), and highlighted us‐GO as the less reactive materials of all three, as previously suggested.^[^ ^38^ ^]^

Altogether, our findings strongly suggest that the primary lateral dimensions of GO sheets, together with their ability to form material agglomerates in the airways, are major determinant factors of their biodistribution and ensuing adverse effects in lungs.

### 3.2 Persistent Granulomatous Response Attenuated by Transformation of GO

Granulomas form when the immune system attempts to wall off a foreign agent that cannot be eliminated.^[^ ^42^ ^]^ This physiological process is for instance used to isolate infectious agents such as *Mycobacterium tuberculosis* in the lungs of infected individuals who will not develop the disease.^[^ ^43^ ^]^ Here, both s‐GO and l‐GO induced the formation of granulomas that persisted for up to 90 days, and this formation appeared to be correlated with the presence of larger material agglomerates for these two materials. Noticeably, the formation of granulomas after l‐GO exposure followed the recruitment of interstitial macrophages and moDCs, which have been previously described in granulomatous response to both sterile and infectious agents,^[^ ^44^, ^45^ ^]^ and are known to stimulate Th1 polarized immune responses during granulomatous inflammation.^[^ ^46^ ^]^ At day 7, the enhanced secretion of both IL‐6 and IFN‐*γ* was also an event favoring a Th1 response to l‐GO. In agreement with those findings, Ma et al. found that large GO sheets (750–1300 nm) induced greater Th1 inflammation in mouse lungs than small GO sheets (50–350 nm).^[^ ^21^ ^]^ Here, l‐GO sheets also inhibited an anti‐inflammatory Th2 immune response by decreasing the expression of IL‐4,^[^ ^47^ ^]^ in agreement with previous works in which GO was shown to attenuate Th2 response in mice.^[^ ^48^ ^]^ Overall, the modulation of the different inflammation mediators observed here after l‐GO exposure was consistent with the sustainment of a pro‐inflammatory granulomatous response.^[^ ^45^ ^]^

However, an important complication of persistent granulomatous inflammation is fibrosis, which may cause permanent tissue remodeling and damages, even after the causative agent has been eliminated.^[^ ^42^ ^]^ In addition, granuloma‐driven fibrosis after pulmonary exposure to carbon nanomaterials has been previously ascribed for inducing loss of lung function and further pathological sequelae, including cancer.^[^ ^49^, ^50^ ^]^ Here, granulomatous inflammation combined with marked fibrosis was only observed in MWCNT (Mitsui‐7) exposed animals, in agreement with previous reports.^[^ ^44^, ^51^ ^]^ Indeed, despite the persistence of lung granulomas after s‐GO or l‐GO exposure for up to 90 days, there was no evidence of fibrosis at any time point. Bengtson et al. reported similar absence of fibrosis for up to 90 days after GO intratracheal instillation in mice, in spite of observing inflammation, acute phase response, or BAL cell genotoxicity.^[^ ^39^ ^]^ In contrast, Li et al. showed that GO materials could induce lung fibrosis as early as 7 days.^[^ ^40^ ^]^ Taken together, these different studies suggest that GO‐induced fibrosis might be GO material specific, even though differences in the delivered dose to the lower airways in these different studies should not be neglected. In the present study, the lack of pulmonary fibrosis despite the formation of granulomas could be explained by a long‐lasting Th1 polarized immune response (combined with an inhibition of pro‐fibrotic Th2 immune response, as mentioned above), and an elevated level of interferons (i.e., IFN‐*β* (ELISA measured, significant at 7 days after l‐GO exposure) or IFN‐*γ* (revealed by analysis of RNA sequencing)), especially after l‐GO exposure. Type I interferons (such as IFN‐*β*) have indeed been associated with the development of chronic inflammation in response to particulate matter by triggering the production of chemokines that further attract inflammatory cells.^[^ ^52^ ^]^ Therefore, which material features and biological mechanisms are specifically associated to the observed differences in different GO materials ability to induce fibrosis should be investigated further.

Besides precluding the development of fibrosis, the sustained recruitment of inflammatory cells especially neutrophils and macrophages, via for instance interferons, could also fulfil a highly beneficial role in terms of GO metabolism and elimination from the lungs. Indeed, previous studies have shown that neutrophils and macrophages could mediate the enzymatic degradation of carbon nanomaterials in mouse lungs, and the fibrotic response was more pronounced in enzyme‐deficient mice when compared to wild‐type mice.^[^ ^53^, ^54^ ^]^ Taken together, these results demonstrated that biodegradation is endogenously alleviating the adverse effects of carbon nanomaterials, including lung fibrosis. In line with these data, Girish et al. provided the first evidence of in vivo degradation of oxidized graphene in several mice organs.^[^ ^55^ ^]^ More recently, we have also demonstrated that GO can be enzymatically degraded in a matter of hours when incubated ex vivo with activated human neutrophils.^[^ ^29^ ^]^ In the present study, changes in the Raman signature of GO present in lung sections were also suggestive of biodegradative processes. In particular, l‐GO suffered pronounced structural changes in the lungs and displayed a gradual but advanced carbon amorphization.

Overall, the lack of fibrosis despite the persistence of granulomas and material agglomerates might hence be explained by the biotransformation of GO materials into less reactive materials, which in turn did not appear to induce pro‐fibrotic Th2 immune response. Therefore, further investigations should focus on the association between biotransformation of GO and Th1/Th2 polarized immune responses, and the role of macrophages in GO degradation.

### 3.3 RNA Sequencing Provides Insights into Pathway of Pulmonary Responses to GO

Transcriptomics approaches afford a considerable degree of sensitivity and are vastly superior to traditional microarray‐based approaches.^[^ ^21^ ^]^ Using RNA sequencing, we found distinct, size‐dependent effects at gene expression level matching the size‐dependent histopathological findings; thus confirming the ability of transcriptomics approaches to detect biologically relevant changes.^[^ ^56^ ^]^ In particular, there was a statistically significant upregulation of MMP12 in mice exposed to l‐GO; this is noteworthy as genetic variants of the gene encoding this protein have been associated with different human lung diseases,^[^ ^57^ ^]^ including emphysema, commonly associated to smoking.^[^ ^58^ ^]^ It may therefore be of interest in future work to study this putative link to lung diseases in the context of chronic GO exposure. Similarly, further analysis of RNA sequencing data revealed that both TNF‐*α* and IFN‐ɣ were central regulators for the affected genes, highlighting the key role of inflammation mediators in the regulation of gene expression in response to l‐GO exposure.

More importantly, several pathways annotated as “cancer‐related” were affected by l‐GO (especially at day 28), but not by s‐GO or us‐GO, including the gene encoding serpin B2, a protein involved in both inflammation and cancer.^[^ ^59^ ^]^ Although these pathways were significantly perturbed, activation scores for each individual gene were not. When putting together granulomas formation and cancer‐related pathways activation, both observed after l‐GO exposure, it is important to consider that the multinucleated macrophages present within granulomas may in fact acquire epithelial cell like features. Indeed, a recent study suggested that granuloma macrophages and pre‐malignant epithelial cells may share common mechanisms of adaptation to chronic genotoxic stress.^[^ ^60^ ^]^ In tuberculosis, granuloma macrophages are also known to adopt an epithelioid appearance, and to undergo reprogramming events involving E‐cadherin‐dependent formation of epithelial‐like cell‐cell junctions.^[^ ^43^ ^]^ Thus, one should be cautious concluding that the present transcriptomics results point toward a definite pro‐carcinogenic potential of l‐GO. In fact, it remains possible that some of the gene expression changes evidenced here are reflective of the granulomatous inflammation or other underlining biological processes sharing commonalities with cancer pathways such as EMT, as previously reported for reduced GO.^[^ ^61^ ^]^ Therefore, more detailed analysis of the potential carcinogenicity of micrometer‐sized GO sheets is warranted. In particular, attention should be paid to comparing the gene expression profiles in granuloma macrophages and in the whole lung tissue.

### 3.4 Considerations Regarding the Exposure Model and Its Implications

The present results highlight some values of i.n. instillation in comparison to or alongside i.t. instillation. Although both methods provide good control of the administered doses, i.t. instillation enables a superior delivery of the nominal dose of materials to the lower respiratory tract. On the other hand, i.n. instillation does not bypass the upper respiratory tract or the mucocilliary clearance, thus providing a more realistic assessment of the ability of materials with varying dimensions to reach the lower airways, and a better evaluation of their impact considering the actual delivered dose. However, material agglomeration in the nasal cavity upon i.n. instillation may affect the delivered dose to the lungs due to potential retention in the cavity, as observed here for l‐GO. Conversely, the nasal cavity of mice has significant anatomical and physiological differences compared to humans, including a much greater surface area of the olfactory epithelium relative to the total mucosal surface.^[^ ^62^ ^]^

In our view, the main limitation of the present study was, however, the delivery of a single dose administered as a bolus; even though this dose was similar to that of several other in vivo studies of GBMs.^[^ ^7^ ^]^ Using the allometric relationships between mice and humans, we estimated that the GO dose used here in mice (50 µg) would correspond to a lung burden equivalent to a human occupational exposure lasting from 2.3 months up to approximately 23.5 years at maximum recommended levels, that is, 50 µg m^−3^ (see calculations in Supporting Information). In another study using gold standard sub‐chronic inhalation exposure of rats, the highest dose deposited in the lungs at aerosol concentration of 9.78 mg m^−3^, was estimated to be 0.515 mg GO (dimensions 0.5–5 µm).^[^ ^36^ ^]^ Following the allometric relationships between rats and mice,^[^ ^70^, ^71^ ^]^ we estimated that this is equivalent to a deposited dose of 64 µg per day in the mouse lungs over a period of 5 days (see calculations in Supporting Information). Therefore, the single bolus dose of 50 µg used here is not excessively high compared to standard inhalation studies. In order to more adequately replicate the type of exposures observed in occupational settings and establish exposure limits for occupational health as well as inform safety risk assessments, it would, however, be important to apply repeated administration of GO, using inhalation or oro‐pharyngeal instillation, in future work.

## 4 Conclusions

The present study demonstrates that lateral dimensions play a fundamental role in the pulmonary response to GO after intranasal instillation in mice. Micrometer‐sized GO induced the strongest adverse response, including granulomas persisting for up to 90 days, despite a reduced translocation to the lungs; whereas animals exposed to nanometer‐sized GO displayed full histological recovery by day 28. Whole tissue molecular analysis of the lung response to the different tested materials confirmed the very distinct and size‐dependent effect of each GO. Importantly, it highlighted some common pathways between cancer development and response to micrometer‐sized GO exposure that require further investigations. Finally, our results revealed that regardless of their dimensions GO sheets while in the lungs are enduring in situ biotransformation leading to a degradation of their crystalline structure, which may partly be ascribed to the absence of lung fibrosis despite the long‐term persistence of both granulomas and material agglomerates in the case of micrometer‐sized GO. Based on our results, it is unlikely that occupational exposure to nanometer‐sized GO (s‐GO and us‐GO) at realistic air concentrations would induce significant pulmonary toxicity. Considering the growing usage of GO or other GBMs in spray coating for anti‐corrosion solutions, we believe the present findings will be essential toward the implementation of a safer‐by‐design approach in the industrial development of GBM enhanced products or applications, for the benefit of workers and end‐users.

## 5 Experimental Section

##### Material Preparation

GO dispersions were prepared following a modified Hummers’ method under endotoxin‐free conditions, as previously described.^[^ ^22^ ^]^ Briefly, the difference in the production of these materials consisted in the application of ultrasounds to break down the GO sheets, after oxidation of graphite flakes. l‐GO was not sonicated, whereas s‐GO and us‐GO underwent sonication steps of 5 min and 4 h in a bath sonicator operating at 80W (VWR, UK) before purification by centrifugation. The morphology (AFM and TEM imaging) and surface properties (XPS analysis) of the three GO materials tested in this study have been previously fully characterized and reported.^[^ ^22^ ^]^ Biodistribution studies were performed after chemical functionalization of GO sheets with NH_2_‐PEG_4_‐DOTA via epoxide ring opening reaction, as previously reported.^[^ ^26^ ^]^ Physicochemical characterization of GO‐DOTA sheets, including labeling stability, demonstrated the maintenance of the oxidation degree and structural properties, making these probes suitable for biodistribution studies. For those studies, GO‐DOTA probes were labeled after chelation of metal isotopes, either the natural ^115^In (Merck‐Sigma, UK) or the radioactive ^111^In (Mallinckrodt, UK), as previously described.^[^ ^26^ ^]^ The MWCNTs (MWCNT‐7, Mitsui‐7) used in this study were kindly provided by Prof Ulla Vogel (National Research Centre for the Working Environment, Denmark, and Technical University of Denmark).

##### Animal Exposures

Six to 8‐week old female C57BL/6 mice (Envigo, UK) were allowed to acclimatize for 7 days prior to any experiment. Animals were kept in groups of five with free access to water and food, under a regular 12 h light/dark cycle between 7 am and 7 pm, at a temperature of 19–22 °C and relative humidity of 45–65%. All procedures were conducted after ethical approval from the UK Home Office, under Project License no. 70/7763, in accordance with the ARRIVE guidelines for animal research.^[^ ^63^ ^]^ Experiments were carried out using four to five animals per group. All GO materials were dispersed in an aqueous solution of 5% (m/v) dextrose in ultrapure water (5% dextrose), at a concentration of 1 mg mL^−1^, about 30 min before administration. MWCNTs were dispersed at the same concentration in an aqueous solution of 0.9% (m/v) sodium chloride supplemented with 0.5% (m/v) bovine serum albumin (BSA), followed by bath sonication for 10 min at 80 W (VWR, UK). Both dispersants were sterile filtered (Merck Millipore, PES membrane, 0.2 µm, 33 mm) prior to use. Animals were anesthesized by inhalation of 2.5% isoflurane in 100% oxygen flowing at 2 L min^−1^. Mice in experimental groups were instilled with 50 µL of the respective nanomaterial dispersions (i.e., 50 µg per mouse), which were equally distributed in each nostril. The same volume of 5% dextrose solution was given to the control group. During administration, the animals were held in a supine position, tilted to about 60°, in order to effectively introduce the full dose. Mice were observed until full recovery, which occurred within 5 min after instillation.

##### Biodistribution Studies

Mice exposed to GO‐DOTA labeled with the natural isotope ^115^In (for ICP‐MS) were dissected 1 and 7 days after i.n. instillation, and organs of interest were extracted and washed in PBS 1x to remove excess blood. Each sample was placed in a small glass vial and dried under a fume hood for several days. Both wet and dry weights were measured using a microbalance (Sartorius MC210S, Goettingen, Germany) prior to tissue solubilization. In the case of GO‐DOTA labelled with radioactive ^111^In (for autoradiography), lungs were extracted 1 day after administration, and only tissue‐dried to remove excess liquid.

*ICP‐MS*. Dried organs were solubilized following a protocol involving multiple rounds of exposure to 1 mL of 30% hydrogen peroxide (Merck‐Sigma Aldrich, UK) at 140 °C until drying, followed by another step using 70% nitric acid (Merck‐Sigma Aldrich, UK). At the end of the solubilization process, each sample was reconstituted with 5 mL of 2% nitric acid. Removal of organic matter was completed by filtering through a 0.2 µm PES membrane (Merck Millipore, UK). Inductively‐coupled plasma mass spectrometry (ICP‐MS) measurements were performed at the Manchester Analytical Geochemistry Unit, University of Manchester, using an Agilent 7500 Series ICP‐MS system (Agilent, UK).

*Autoradiography*. Deposition of GO‐DOTA in the lungs was investigated by chelating with ^111^In. As a vehicle control, mice were exposed to the DOTA[^111^In] probe. All probes were administered with similar radioactivity (5.5–6.5 MBq), corresponding to the same volume of instilled dose. Extracted lungs were fixed to two autoradiography cassettes (Kodak Biomax Cassette) using transparent sticky tape, and left in contact with super sensitive plates (Cyclone storage phosphor screen, Packard) for 3 days. Autoradiographs were scanned using a cyclone phosphor detector (Packard Biosciences, UK) and processed using OptiQuant software (version 3.1, Packard Bioscience). Signal intensity scales were matched to take into account minor differences in background of the two cassettes.

##### Sample Collection

Mice were sacrificed at days 1, 7, 28, or 90 days after exposure by overdose via intraperitoneal injection of 0.2 mL pentobarbitone. Except at day 90, BAL was performed by injecting the airways with 1.5 mL of Hank's balanced salt solution (HBSS, Gibco, Thermo Fisher Scientific, UK), which were slowly flushed up and down prior to extraction to a 1.5 mL microcentrifuge tube, which was immediately placed on ice until processing. At all considered time points, the lungs were carefully dissected from the large airways and small portions of each lobe were extracted for RNA extraction. Tissue samples for protein analysis were also collected at days 1 and 7 post exposure. Tissue samples were placed in freshly prepared RIPA buffer or RNAlater (Invitrogen, Thermo Fisher Scientific, UK), respectively, and placed immediately on ice. Samples in RNAlater solution were incubated overnight at 4 °C prior to storage at −20 °C, until further processing. The remaining lung tissue was fixed in 4% paraformaldehyde (PFA) overnight at 4 °C. The left lung was transferred to a solution of 70% ethanol before paraffin embedding for histology, while the right lung was cryopreserved in 30% (m/v) sucrose dissolved in ultrapure water before snap‐freezing in optical cutting temperature (OCT) medium for cryo‐sectioning.

##### Histopathology

Paraffin‐embedded lung samples were sectioned using a Leica RM2255 microtome set at a thickness of 5 µm. Hematoxylin and eosin or Masson's trichrome staining was employed to measure pathological changes and collagen deposition, respectively. Images were collected using a Pannoramic 250 Flash slide scanner (3D Histech, Hungary), in bright‐field mode, and analyzed using Pannoramic Viewer (version 1.15.4, 3D Histech, Hungary). Areas of evident interstitial infiltration, characterized by alveolar wall thickening and granuloma formation, were manually segmented using ImageJ software (version 1.51, National Institutes of Health, Bethesda, MD). Cell infiltration was defined as the sum of these manually segmented areas divided by the total area of the lung section. Infiltrate size corresponded to the area of each segmented area.

##### Raman Mapping

Lung samples snap‐frozen in OCT were sectioned using a Leica CM3050S cryotome set at a thickness of 20 µm. Cryo‐sections of lungs exposed to GO materials were gently rinsed with PBS 1x, ultrapure water, and 100% methanol, in order to remove any excess of OCT compound and salts, and to post‐fix the section. Once dried, lung sections were imaged under an Olympus BX41 microscope, part of the DXRxi Raman system (Thermo Scientific, UK), using a 50x objective. Raman spectra were acquired using a laser of *λ* = 633 nm operating at 0.4 mW, through a 50 µm pinhole aperture with an exposure time of 0.125 s. Raman correlation maps were plotted using the OMNICxi software (Thermo Scientific, UK), in order to reflect the spatial distribution and transformation of GO in the lungs. An arbitrary color scale was defined at each pixel to describe the similarity between the acquired Raman spectrum at that coordinate and a reference spectrum of GO, focusing on a region comprising both the D and G bands (1145–1810 cm^−1^). Black color represents background signal, whereas white color represents the highest similarity to the reference spectrum, indicating the presence of GO. Red color represents a weaker signal of the D and G bands compared to the reference spectrum.

##### BAL Analysis

BAL fluid samples were centrifuged at 1500 rpm (214 g) for 5 min at 4 °C using a Hettich Universal 320R centrifuge (Hettich Zentrifuger, Germany). Supernatant was collected for the quantification of total protein and amount of released lactate dehydrogenase (LDH), whereas the pellet was re‐suspended in 0.5 mL of PBS for differential cell counting.

*BCA Assay*. Protein release in BAL fluid was quantified using the bicinchoninic acid (BCA) protein assay (Pierce, Thermo Fisher Scientific, UK), according to the manufacturer's instructions. Briefly, 200 µL of BCA reagent mixture were added to 25 µL of each sample, and incubated at 37 °C for 30 min in a 96‐well plate (Corning, UK). Optical absorbance at 562 nm was measured using a FLUOstar Omega plate reader (BMG Labtech, UK). The protein concentration of each sample was determined via extrapolation from the BSA standard curve, defined with albumin standards (0–2000 µg mL^−1^). Standard solutions and BCA reagent mixture were freshly prepared before each assay.

*LDH Assay*. LDH content was measured using the CytoTox 96 Non‐Radioactive Cytotoxicity Assay (Promega, UK). In this case, 50 µL of each sample were mixed with 50 µL of LDH substrate mix, followed by incubation in the dark for 15 min at room temperature. After adding 50 µL of stop solution, optical absorbance at 490 nm was measured using a FLUOstar Omega plate reader (BMG Labtech, UK).

*Differential Cell Counting*. Cells in BAL fluid were quantified using a haemocytometer after Trypan Blue exclusion staining. Live cells that did not internalize the dye were visualized under a PrimoVert inverted microscope (Carl Zeiss, UK) using a 20x objective. Cell suspensions underwent a cyto‐centrifugation step at 600 rpm (34 g) for 5 min at 4 °C, followed by fixation in ice‐cold 100% methanol for 10 min. Differential cell staining was performed using the Kwik‐Diff kit (Thermo Fisher Scientific, Shandon, UK) according to the manufacturer's protocol. Stained cells were imaged under an Olympus BX41 microscope (Olympus, UK).

##### Flow Cytometry

In a separate experiment, mice were intranasally instilled with carbon nanomaterials and sacrificed 1 and 7 days post exposure. Lungs were carefully dissected from the large airways and split into small portions using scissors. These lung portions were transferred to tubes containing 2 mL of digestion buffer, comprised by Liberase‐TM (Roche, UK) at a concentration of 0.4 Wünsch units mL^−1^ and DNase I (Roche, UK) at a concentration of 0.01 mg mL^−1^, in RPMI‐1640 cell culture medium (Life Technologies, Thermo Fisher Scientific, UK), supplemented with 10% fetal bovine serum (FBS, Gibco, Thermo Fisher Scientific, UK). After shaking for 30 min at 37 °C, the digested lungs were filtered through a 100 µm mesh filter and supplemented with cell culture medium with 5 mm EDTA. The resulting cell suspension underwent lysis of red blood cells using Red Blood Cell Lysing Buffer Hybri‐Max (Merck‐Sigma Aldrich, UK) before centrifugation at 500 g for 7 min to remove cell debris. Obtained cell suspensions were quantified by Trypan blue exclusion before staining for flow cytometry. The staining protocol involved the incubation with the Live/Dead UV viability dye (Life Technologies, Thermo Fisher Scientific, UK) for 20 min at room temperature, before washing and staining with a mixture of Fc blocking agent (anti‐mouse CD16/CD32) and fluorochrome‐conjugated antibodies (see Table S4, Supporting Information) for 30 min at 4 °C. Cells were finally fixed with 2% PFA in cell culture medium for 20 min at room temperature, and washed prior to flow cytometry measurements. Data were acquired using a BD Fortessa flow cytometer (BD Biosciences, UK), and then processed using FlowJo (version 10, FlowJo, LLC, USA). Appropriate single‐color and fluorescence minus one (FMO) controls were performed for all fluorochromes, in order to compensate any spectral overlap and potential non‐specific binding and/or fluorescence emission in undesired channels. Cell populations were defined as shown in Figure S10, Supporting Information. Cell events were gated after excluding cell debris and doublets, using both forward (FSC) and side scatter (SSC). Leukocytes were defined by selecting CD45^+^ cells that had low fluorescence intensity for the Live/Dead signal. Neutrophils were discriminated based on their high expression of Ly6G and CD11b, and confirmed by their intermediate SSC values, indicating their granularity. The remaining cells were gated based on their expression of Siglec‐F. Siglec‐F^+^ cells were distinguished based on their expression of CD11b, with eosinophils expressing CD11b and having high SSC. Alveolar macrophages, on the other hand, were CD11b^−^ and confirmed to be CD11c^+^ and CD64^+^. Myeloid cells were further gated based on their expression of CD11b and CD11c. Dendritic cells (DCs) were considered CD11c^+^ cells, and based on their expression of CD11b, Ly6C and MHC‐II they could be further classified: i) conventional DCs (cDCs) were considered CD11b^−^ CD11c^+^ Ly6C^−^ MHC‐II^+^; ii) plasmacytoid cells (pDCs) could be found in the CD11b^−^ CD11c^+^ Ly6C^+^ MHC‐II^−/int^ population; iii) CD11b^+^ DCs differed from cDCs due to their expression of CD11b (CD11b^+^ CD11c^+^ Ly6C^−^ MHC‐II^+^); and iv) monocyte‐derived DCs (moDCs) were CD11b^+^ CD11c^+^ Ly6C^+^ MHC‐II^−/int^. Monocytic cells were gated on the CD11b^+^ CD11c^−^ population, with interstitial macrophages expressing CD64. Monocytes were divided into classical (Ly6C^+^) or patrolling (Ly6C^−^), depending on their activation state. Lymphocytes were identified as CD3^+^, from the population of CD11b^−^ CD11c^−^ cells, and further classified either as CD4^+^ or CD8^+^ T cells. The obtained percentages for each cell population were multiplied by the number of live cells after Trypan blue exclusion, rendering absolute cell counts.

##### Cytokine Analysis

Tissue incubated in RIPA buffer was homogenized with 5 mm stainless steel beads using a TissueLyser LT system (QIAGEN, UK) operating at 50 Hz for 5 min, followed by centrifugation at 2600 g for 5 min to remove cell debris. The obtained supernatant was utilized in subsequent protein analysis. Protein concentration was determined using the BCA assay, as described above. Quantification of a set of inflammatory cytokines and chemokines (IL‐1*α*, IL‐1*β*, IL‐6, IL‐10, IL‐12p70, IL‐17A, IL‐23, IL‐27, MCP‐1, IFN‐*β*, IFN‐*γ*, TNF‐*α*, and GM‐CSF) was carried out using a LEGENDplex Mouse Inflammation kit (BioLegend, UK) according to the manufacturer's recommendations. Fluorescence intensities of the multiple analytes were recorded using a BD FACSCanto flow cytometer (BD Biosciences, UK). Data analysis was performed with specialized software provided by the manufacturer of the assay Cytokine concentration was normalized by total protein concentration as determined above.

##### RNA Extraction and RT‐qPCR

Tissue samples in RNAlater solution (Invitrogen, Thermo Fisher Scientific, UK) were homogenized using a TissueLyser LT (QIAGEN, UK) as described above. Homogenized samples were then loaded onto spin cartridges containing silica membranes, for extraction of total RNA performed using a PureLink RNA Mini kit (Invitrogen, Thermo Fisher Scientific, UK) according to the manufacturer's instructions. Total RNA concentration and purity were calculated by measuring the optical density at 230, 260, and 280 nm, using a Biophotometer Plus spectrophotometer (Eppendorf AG, Germany). For quality control purposes, RNA samples were required to have absorbance ratios at 260 nm/280 nm and 260 nm/230 nm above 1.8. First‐strand cDNA was created from 1 µg of RNA in a total volume of 20 µL using the High Capacity cDNA Reverse Transcription kit (Invitrogen, Thermo Fisher Scientific, UK). The cDNA synthesis reaction followed a protocol of 10 min at 25 °C, followed by 2 h at 37 °C, and 5 min at 85 °C, executed by a CFX96 real‐time PCR system (BioRad, UK). Quantitative PCR was performed using the CFX96 real‐time PCR detection system (BioRad, UK). Each sample consisted of 2 µL of cDNA obtained from reverse transcription, each primer at 500 nm, and 10 µL of PowerUp SYBR Green Master Mix (Applied Biosystems, Thermo Fisher Scientific, UK) in a 20 µL reaction. Primers are listed in Table S5, Supporting Information. PCR protocol consisted of an initial activation step at 50 °C for 2 min, followed by a denaturation step at 95 °C for 2 min, prior to 40 cycles of amplification (denaturation at 95 °C for 15 s, then annealing and elongation at 60 °C for 1 min). Melting curves between 65 and 95 °C were also obtained, in order to confirm amplification specificity and the absence of artefacts such as primer dimers. No‐template controls did not return any Ct values. Each sample was analyzed in duplicate, and the mean Ct value was used for further calculations. Relative changes of gene expression of each treatment were calculated according to the Livak method as 2^−ΔΔCt^, where the obtained ΔCt values are subtracted by those obtained with the negative control (Dextrose). The ΔCt values were calculated by subtracting the Ct value of each individual gene by the Ct value of the housekeeping gene in each sample, in order to normalize the amount of each transcript.

##### RNA Sequencing

Total RNA from tissue sample was extracted as described above. RNA quantity and RNA integrity number (RIN) were assessed by Agilent 2200 bioanalyzer. Sample RNA with RIN values below 8 was discarded. The isolated RNA samples were stored at −80 °C until RNA sequencing was carried out. Illumina TruSeq Stranded mRNA Library Prep Kit (Illumina, Inc., San Diego, CA) was used to prepare mRNA sequencing libraries. Briefly, 1000 ng of total RNA was used for mRNA isolation using poly dT‐coated beads. After purification, the mRNA was chemically fragmented into small pieces, which was subsequently used as a template for cDNA synthesis using reverse transcriptase followed by short fragment removal from another purification step. After end‐repair, adapter ligation, and index codes adding for each sample, PCR amplification was conducted. Unaligned adapters were removed twice after adapter ligation and PCR amplification. The quality of the libraries were examined by Caliper LabChip GX/HT DNA high sensitivity and the quantitation of libraries were measured by Qubit dsDNA HS. All 36 libraries were paired‐end sequenced on a HiSeq2500 (HiSeq Control Software 2.2.58/RTA 1.18.64) with a 2 × 126 setup using “HiSeq SBS Kit v4” chemistry. The conversion from Bcl to FastQ was performed using “bcl2fastq2 v2.19” from the CASAVA software suite. The quality scale used is Sanger/phred33/Illumina 1.8+.

##### Bioinformatics Analysis

The quality check involving the analysis of sequence quality, GC content, the presence of adaptors, overrepresented sequences, duplication level in order to detect sequencing errors, PCR artifacts or adapter contaminations was conducted using software tools FastQC. Fastq‐Screen was performed to check the composition of the libraries. The mRNA sequencing data were analyzed using the rnaseq pipeline (https://github.com/nf‐core/rnaseq). The preprocessed sequencing reads were aligned against the *Mus musculus* (GRCm38) reference genome using STAR.^[^ ^64^ ^]^ Subsequently, the read‐counts to genes were generated using the software featureCounts.^[^ ^65^ ^]^ Full‐length transcripts representing multiple splice variants for each gene locus were assembled and quantitated using StringTie.^[^ ^66^ ^]^ Extensive quality‐control on the results was performed using RSeQC,^[^ ^67^ ^]^ dupRadar,^[^ ^68^ ^]^ and Preseq (http://smithlabresearch.org/software/preseq/) which generated RNA quality control metrics, technical/biological read duplication level, and library complexity estimation. Differentially expressed genes between different treatments of samples were determined using edgeR.^[^ ^69^ ^]^ Differential gene expression levels between the healthy control lung tissue sample and the three different GOs (us‐GO, s‐GO, l‐GO) were estimated with a *t*‐test. *p* values were corrected with the Benjamini–Hochberg algorithm (false discovery rate; FDR). DEGs having a fold change (logFC) ≥ 0.5, *p*‐value ≤ 0.05 were considered for functional analysis. Ingenuity Pathway Analysis (application version 220 217, content version 16 542 223) (licensed by Ingenuity Systems, Redwood City, CA) was employed to study biological processes, canonical pathways, upstream transcriptional regulators, and gene networks, based on the DEGs.

##### Statistical Analysis

All experiments were analyzed using GraphPad Prism software (version 6.01, GraphPad Inc., USA). Data were analyzed using analysis of variance (two‐way ANOVA) with post hoc Sidak's multiple comparisons test or Kruskal–Wallis test with post hoc Dunn's multiple comparisons test. In both cases, *p* < 0.05 was considered significant.

## Conflict of Interest

The authors declare no conflict of interest.

## Author Contributions

A.F.R. implemented the experiments and analyzed the data, under the supervision of C.B. and K.K. L.N. and D.A.J. performed biodistribution experiments. I.A.V., C.M.M., and A.B. prepared the functionalized nanomaterials for biodistribution experiments. S.P.M. performed R.N.A. sequencing and the bioinformatics analysis, while J.W. performed statistical analysis of sequencing data, both under the supervision of B.F. A.F.R., K.K., and C.B. conceived the overall design of the study. A.F.R., C.B., and B.F. wrote the manuscript, which was revised by all authors.

## Supporting information

Supporting Information
