# Supplementary material for: Size‐Dependent Pulmonary Impact of Thin Graphene Oxide Sheets in Mice: Toward Safe‐by‐Design
Source: Adv Sci (Weinh). 2020 May 7;7(12):1903200. doi: 10.1002/advs.201903200 (PMC7312279; doi:10.1002/advs.201903200)
Supplement: Supplementary file 1 — Supporting Information [file ADVS-7-1903200-s001.pdf]

Supporting Information

Size-dependent pulmonary impact of thin graphene oxide sheets in mice: towards safe-by-design

Artur Filipe Rodrigues[1,2,3], Leon Newman[1,2], Dhifaf Jasim[1,2], Sourav P. Mukherjee[4], Jun Wang[5], Isabella A. Vacchi[6], Cécilia Ménard-Moyon[6], Alberto Bianco[6], Bengt Fadeel[4#], Kostas Kostarelos[1,2#], and Cyrill Bussy[1,2,3,#]

SUPPLEMENTARY TABLE LEGENDS

Table S1. Summary of GO materials characteristics (data reproduced from [22]). *N.D. not detectable.* Values are reproduced from Rodrigues et al 2018 2D Mater.5 035020;

https://doi.org/10.1088/2053-1583/aac05c

Material	Average Lateral Dimensions	Thickness (AFM)	C:O ratio (XPS)	Functionalisation degree (TGA)	Endotoxin level
l-GO	1 µm – 30 µm	1 -7 nm	2.2	37.0%	N.D.
s-GO	50 nm – 2 µm	1 -3 nm	2.1	39.1%	N.D.
us-GO	10 nm – 300 nm	1- 2 nm	2.2	36.7%	N.D.

Values are reproduced from Rodrigues et al 2018 2D Mater.5 035020; https://doi.org/10.1088/2053-1583/aac05c

Table S2. Biodistribution of GO after intranasal instillation. Relative instilled dose (%ID) was quantified by ICP-MS. Values correspond to mean ± SD.

	Day 1				Day 7			
Organ	l-GO	s-GO	us-GO	DOTA	l-GO	s-GO	us-GO	DOTA
Lungs	7.01 ± 4.42	17.48 ± 7.44	12.08 ± 5.77	2.47 ± 1.48	8.25 ± 3.09	5.76 ± 3.12	12.25 ± 1.77	1.73 ± 0.26
Trachea	0.74 ± 0.35	2.61 ± 1.00	1.19 ± 0.58	0.49 ± 0.31	1.19 ± 0.85	0.40 ± 0.40	1.27 ± 0.54	0.22 ± 0.27
Nasal Cavity	1.59 ± 0.97	0.78 ± 0.17	0.54 ± 0.23	0.95 ± 0.24	0.54 ± 0.72	0.84 ± 0.81	1.20 ± 0.74	0.39 ± 0.40
Brain	0.47 ± 0.29	0.74 ± 0.71	0.71 ± 0.54	0.42 ± 0.18	0.27 ± 0.23	0.27 ± 0.25	2.80 ± 1.52	0.36 ± 0.59
GI Tract	2.12 ± 0.99	1.28 ± 1.03	1.23 ± 0.27	0.27 ± 0.19	0.31 ± 0.08	0.26 ± 0.12	0.44 ± 0.25	0.33 ± 0.49
Spleen	0.08 ± 0.08	0.72 ± 1.28	0.21 ± 0.09	0.04 ± 0.02	0.57 ± 0.55	0.65 ± 0.73	0.52 ± 0.83	0.21 ± 0.22
Liver	1.04 ± 0.22	1.05 ± 0.36	1.21 ± 0.64	0.36 ± 0.18	0.77 ± 0.64	0.15 ± 0.07	0.37 ± 0.31	0.54 ± 1.01
Kidney	0.22 ± 0.03	0.57 ± 0.27	0.21 ± 0.03	0.16 ± 0.09	0.19 ± 0.15	0.23 ± 0.15	0.38 ± 0.12	0.32 ± 0.35

Table S3. GO uptake by different organs after intranasal instillation. Amount of instilled dose was normalised by dry tissue weight (mg/g). Values correspond to mean ± SD.

	Day 1				Day 7			
Organ	l-GO	s-GO	us-GO	DOTA	l-GO	s-GO	us-GO	DOTA
Lungs	11.88 ± 6.38	30.72 ± 13.55	20.93 ± 6.37	4.63 ± 2.52	12.62 ± 5.90	8.23 ± 4.46	21.00 ± 3.74	3.20 ± 0.96
Trachea	2.82 ± 1.10	4.52 ± 1.71	3.71 ± 1.31	1.15 ± 0.73	3.30 ± 2.27	1.53 ± 1.19	5.22 ± 1.03	0.50 ± 0.51
Nasal Cavity	11.25 ± 4.17	4.17 ± 1.02	2.04 ± 1.10	4.30 ± 1.49	3.19 ± 3.29	3.24 ± 1.31	5.97 ± 3.15	2.01 ± 1.96
Brain	0.23 ± 0.12	0.39 ± 0.37	0.39 ± 0.24	0.24 ± 0.14	0.14 ± 0.11	0.14 ± 0.13	1.59 ± 0.85	0.20 ± 0.32
GI Tract	0.16 ± 0.06	0.11 ± 0.10	0.09 ± 0.02	0.02 ± 0.01	0.03 ± 0.01	0.02 ± 0.00	0.04 ± 0.03	0.03 ± 0.04
Spleen	0.28 ± 0.26	1.86 ± 3.15	0.93 ± 0.65	0.11 ± 0.07	1.54 ± 1.33	1.10 ± 1.24	1.49 ± 1.96	0.52 ± 0.56
Liver	0.19 ± 0.04	0.23 ± 0.09	0.24 ± 0.12	0.08 ± 0.04	0.11 ± 0.07	0.03 ± 0.02	0.06 ± 0.03	0.10 ± 0.19
Kidney	0.15 ± 0.02	0.56 ± 0.31	0.27 ± 0.11	0.12 ± 0.06	0.18 ± 0.15	0.23 ± 0.20	0.35 ± 0.14	0.27 ± 0.29

Table S4. Antibody-drug conjugates used to stain lung cells for flow cytometry.

Antigen	Fluorophore	Clone	Supplier	λ_{exc} / λ_{em} (aperture)	Dilution
Ly6G	FITC	1A8	BioLegend	488/530 (30)	1:100
CD11b	PE-Cy7	M1/70	BioLegend	561/780 (60)	1:200
Ly6C	eF450	HK1.4	eBioscience	405/450 (50)	1:200
CD11c	BV650	N418	BioLegend	405/660 (20)	1:200
CD45	BV510	30-F11	BioLegend	405/525 (50)	1:400
CD3	APC-Cy7	17A2	BioLegend	640/780 (60)	1:200
CD4	APC	GK1.5	BioLegend	640/670 (14)	1:200
CD8	PerCP-Cy5.5	53-6.7	BioLegend	488/710 (50)	1:200
CD64	PE	X54-5/7.1	BioLegend	561/586 (15)	1:200
Siglec F	PE-CF594	E50-2440	BD Bioscience	561/610 (20)	1:200
MHC II	BV785	M5/114.15.2	BioLegend	405/780 (60)	1:500
Fc Block (CD16/CD32)		93	eBioscience		1:100
Live/Dead	UV		Life Technologies	355/450 (50)	1:2000

Table S5. Primer sequences used for quantifying gene expression by RT-qPCR.

Gene	Accession no. (GenBank)	Primer	Sequence (5' → 3')
Il4	NM_021283.2	Forward	GAGACTCTTTCGGGCTTTTC
		Reverse	TGATGCTCTTTAGGCTTTCCA
Tgfb1	NM_011577.2	Forward	AACTATTGCTTCAGCTCCACAGAG
		Reverse	GTTGGCATGGTAGCCCTTG
Gadph	NM_008084.3	Forward	TGGCCTACATGGCCTCCA
		Reverse	TCCCTAGGCCCCTCCTGTTAT

SUPPLEMENTARY FIGURE LEGENDS

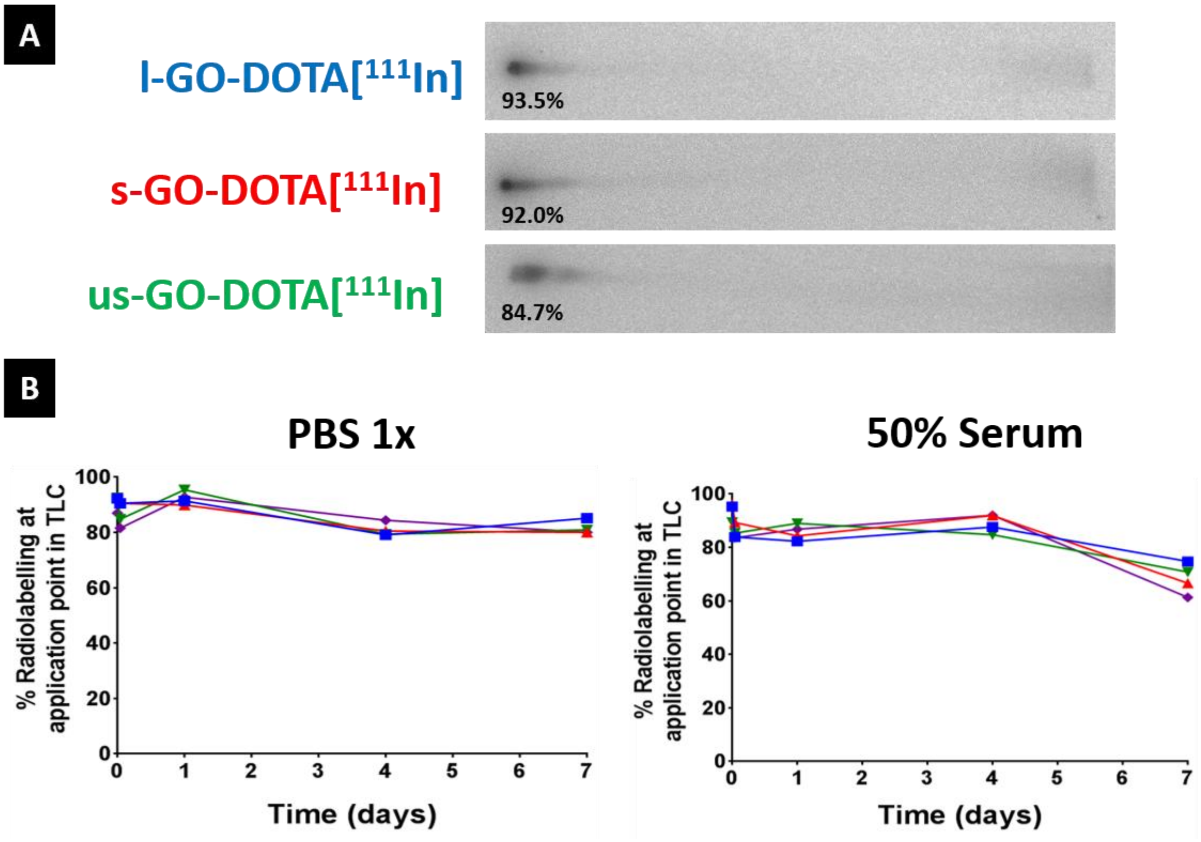

Figure S1. Radiolabelling purity and stability of GO-DOTA[^{111}In]. (A) Radiolabelling purity of probes was determined prior to intranasal administration using thin layer chromatography, in order to compare labelling efficiency of GO-DOTA complexes. **(B)** Stability in physiological media such as 50% serum or PBS was determined *in vitro*, after incubating dispersions for up to 7 days at a temperature of 37°C. All GO-DOTA probes exhibited high radiolabelling stability in physiological media.

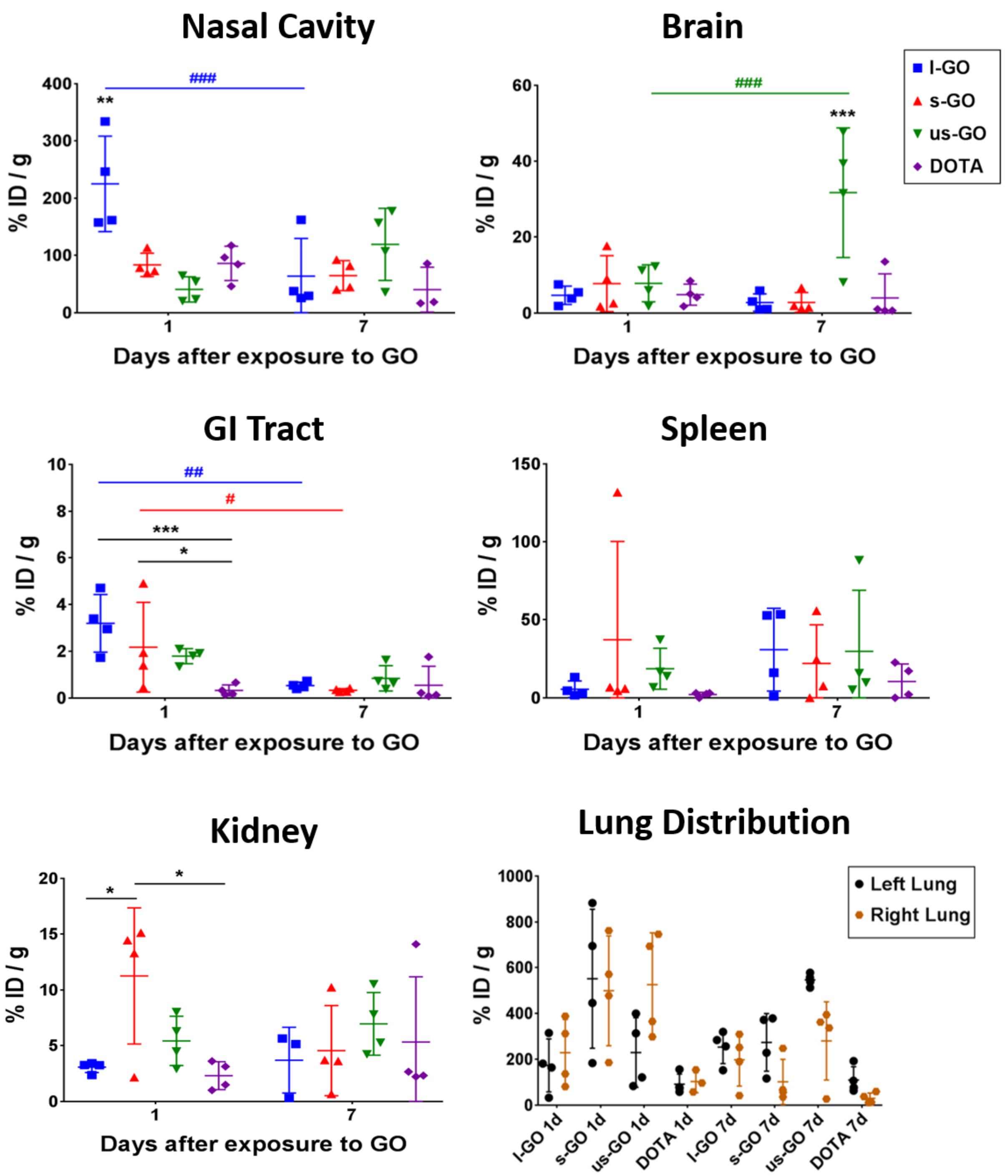

Figure S2. Biodistribution of GO-DOTA[^{115}In] after intranasal instillation. Mice were instilled with DOTA-functionalised GO sheets labelled with naturally occurring ^{115}In, and organs were collected 1 and 7 days after exposure. Quantification of ^{115}In by ICP-MS in relevant extra-pulmonary organs revealed size-dependent clearance profiles, with l-GO being noticeably found in nasal cavity and GI tract, whereas s-GO translocated to the kidney and

us-GO translocated to the brain. No significant accumulation in the reticuloendothelial system was detected at the considered time points. Moreover, amount of GO sheets in each lung lobe was quantified to assert whether their distribution in the lungs was homogenous. No significant differences were detected between left and right lungs, although patterns of accumulation appeared to be random. Individual data points corresponding to each animal are plotted alongside mean values ± SD (n = 4). Data are presented as % instilled dose (ID) per gram of dry tissue. Data were analysed using a 2-way ANOVA test with *post hoc* Sidak's multiple comparisons test. Significant differences between treatments are plotted with (*), whereas differences over time are plotted with (#). In both cases, statistical significance is reported as: **(*)**, $p < 0.05$; **(**)**, $p < 0.01$; **(***)**, $p < 0.001$.

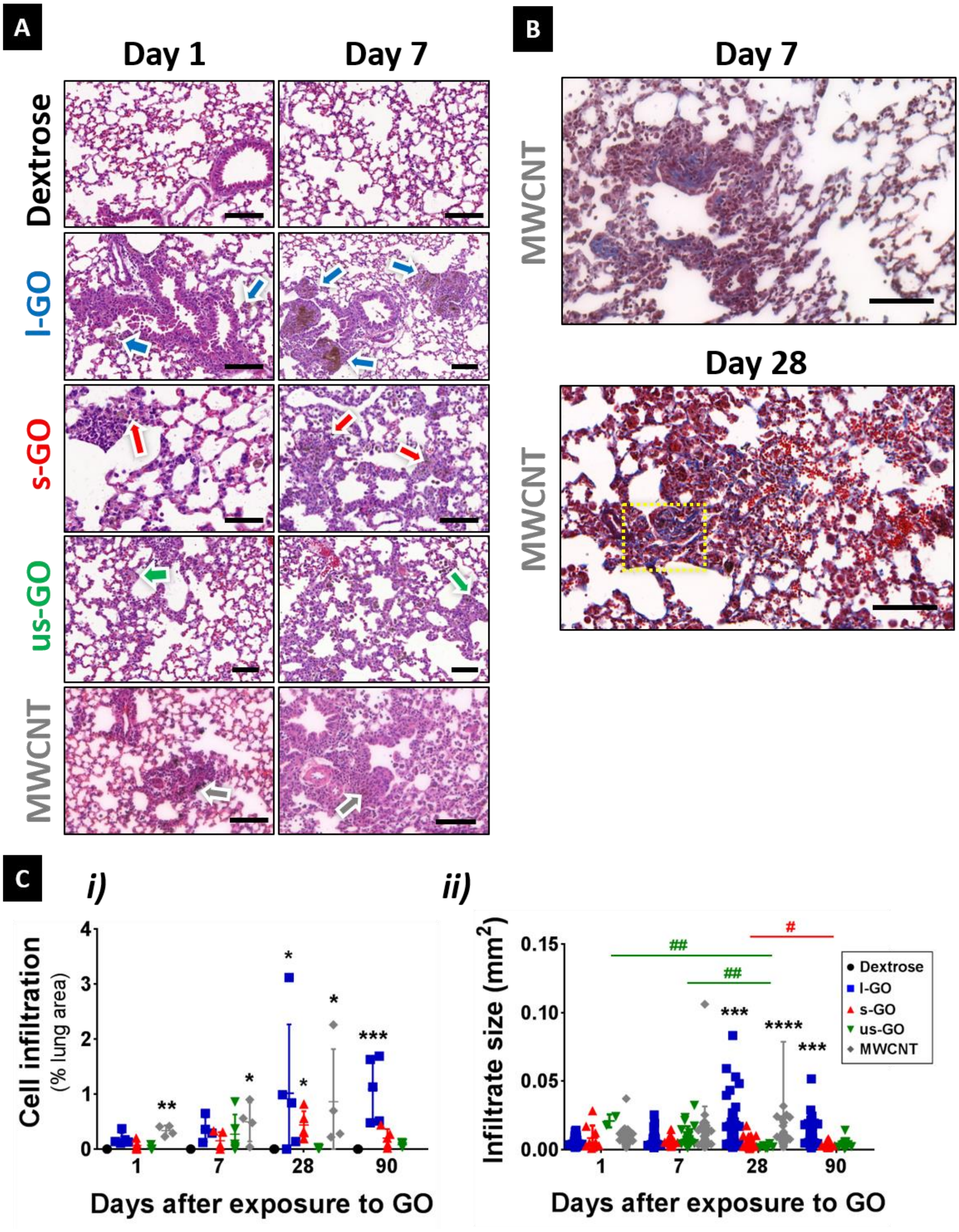

Figure S3. Pulmonary response to MWCNTs after intranasal instillation. Lung sections from mice instilled with GO or MWCNTs were extracted at 1, 7, and 28 days after i.n. instillation. **(A)** Representative images of sections stained with H&E at different time points reveal the development of granulomatous inflammation to l-GO and s-GO, whereas us-GO

only induced a mild acute response. Arrows indicate areas of significant immune cell infiltration in response to the presence of GO, with alveolar wall thickening and granuloma formation. Scale bars = 100 µm. **(B)** Masson's trichrome staining illustrates pro-fibrotic responses to MWCNTs, as demonstrated by collagen deposition in the lung interstitium. Area highlighted with a yellow box corresponds to magnified image presented in **Figure 2**. Scale bars = 100 µm. **(C)** Histopathological analysis of granulomatous response was performed by manually segmenting areas of interstitial inflammation using ImageJ software: **i)** Pulmonary inflammation was quantified as the ratio between the sum of those areas and the total area of the lung section (% infiltration); **ii)** Infiltrate size of each segmented inflamed area was plotted by treatment. Individual data points alongside mean ± SD are presented (n = 4-5 animals/group). Statistical analysis was performed using a Kruskal-Wallis test with *post hoc* Dunn's multiple comparisons test against the negative control (dextrose): **(*)**, $p < 0.05$; **(***)**, $p < 0.001$; **(****)**, $p < 0.0001$. The temporal variation of interstitial inflammation in the lungs was also analysed using a Kruskal-Wallis test with *post hoc* Dunn's multiple comparisons test: **(#)**, p < 0.05; **(##)**, $p < 0.01$.

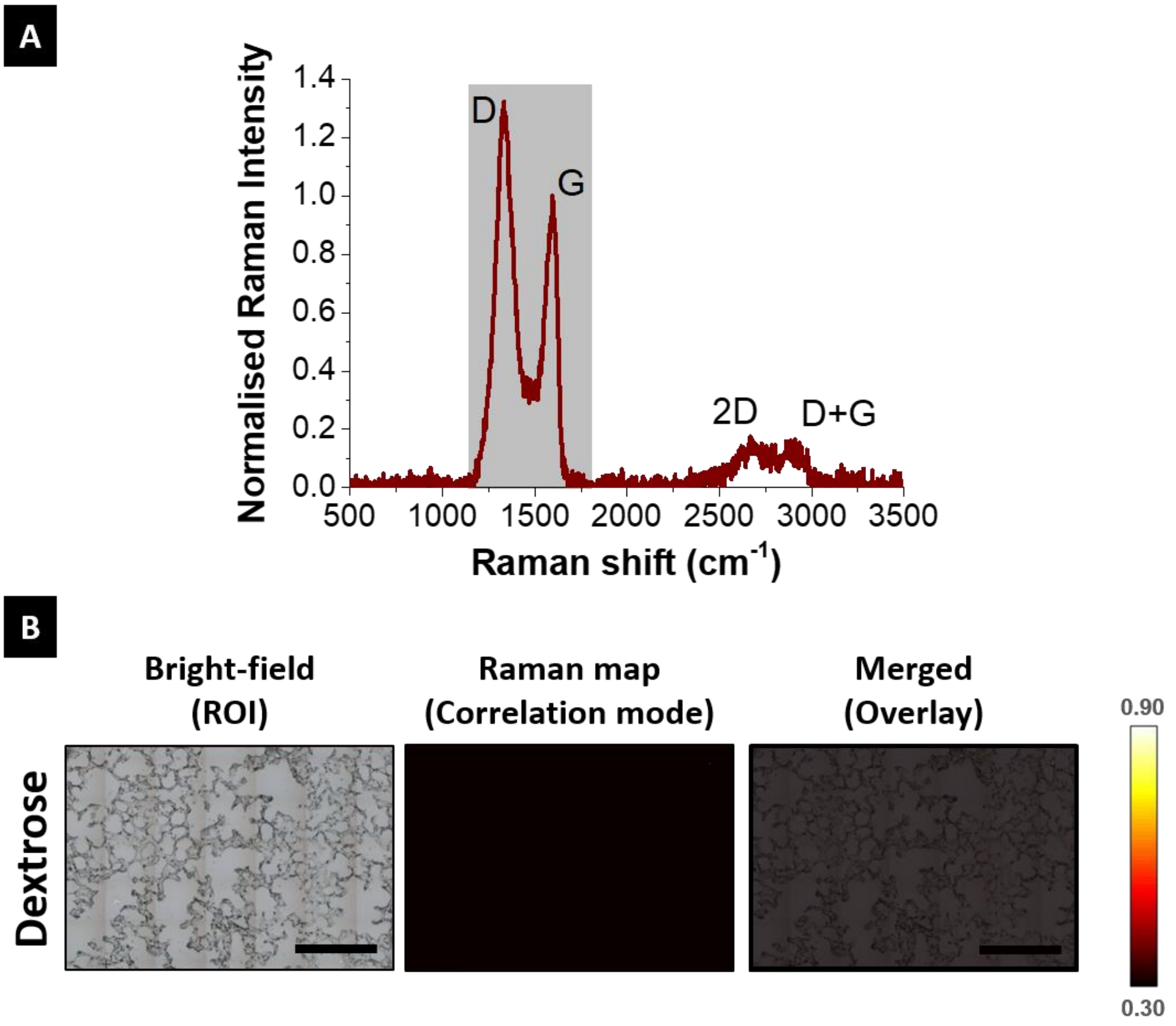

Figure S4. Controls for Raman spectroscopy-based imaging of GO in lung tissue. (A) Reference Raman spectrum of GO. **(B)** Raman spectra were acquired on sections of lungs from mice exposed to GO. Raman mapping analysis compares each acquired individual spectrum for each pixel of the map with this specific reference spectrum. Correlation was performed within a fixed region between 1145 and 1810 cm^{-1} (highlighted in grey), in order to exclude the interference of background signal from the tissue and emphasise the presence of the D and G bands. Raman mapping of lung sections from mice exposed to 5% dextrose did not evidence any sign of Raman-positive areas corresponding to the presence of GO. Scale bars = 200 µm.

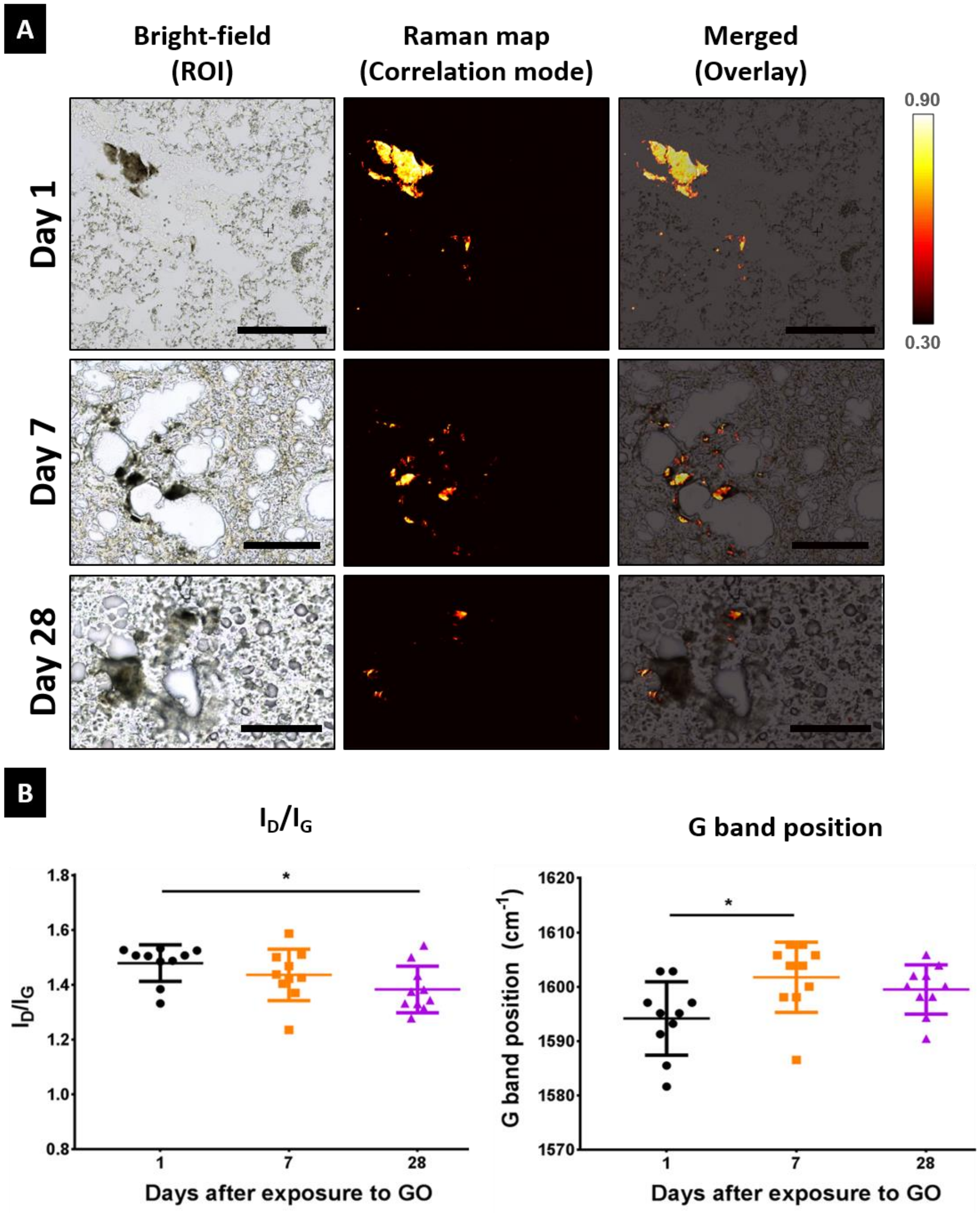

Figure S5. Raman mapping of lung sections from mice exposed to l-GO. Lungs were harvested from mice exposed to l-GO at days 1, 7 and 28 post exposure. **(A)** Raman spectroscopy revealed large areas of strong correlation indicating the presence of GO, as shown by the correlation maps overlaying the region of interest (ROI) in bright-field images.

The decreased correlation at days 7 and 28 suggests the occurrence of clearance and/or material biotransformation. Scale bars = 200 µm. **(B)** Evolution of the structure of l-GO in the lungs was assessed by calculating the ratio between the intensities of the D and G bands (I_D/I_G). Position of the G band was also evaluated to determine the emergence of the D' band. Individual data points correspond to 10 acquired spectra obtained from Raman maps, alongside mean values ± SD. Statistical analysis was performed using a Kruskal-Wallis test with *post hoc* Dunn's multiple comparisons test: **(*)**, $p < 0.05$.

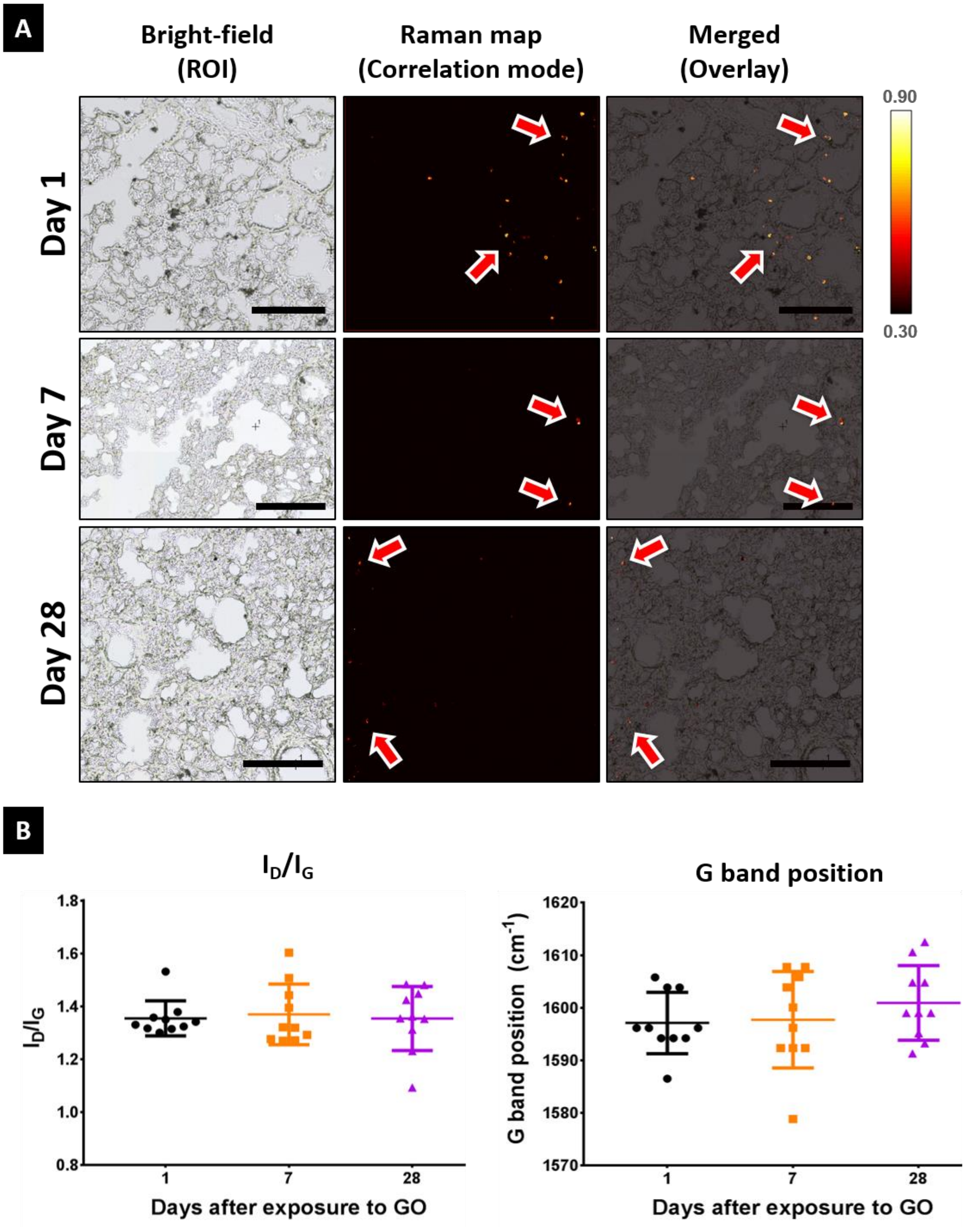

Figure S6. Raman mapping of lung sections from mice exposed to s-GO. Lungs were harvested from mice exposed to l-GO at days 1, 7 and 28 post exposure. **(A)** Raman spectroscopy revealed large areas of strong correlation indicating the presence of GO, as shown by the correlation maps overlaying the region of interest (ROI) in bright-field images.

The decreased correlation at days 7 and 28 suggests the occurrence of clearance and/or material biotransformation. Scale bars = 200 µm. **(B)** Evolution of the structure of s-GO in the lungs was assessed by calculating the ratio between the intensities of the D and G bands (I_D/I_G). Position of the G band was also evaluated to determine the emergence of the D' band. Individual data points correspond to 10 acquired spectra obtained from Raman maps, alongside mean values ± SD. Statistical analysis was performed using a Kruskal-Wallis test with *post hoc* Dunn's multiple comparisons test. No statistical significance was obtained.

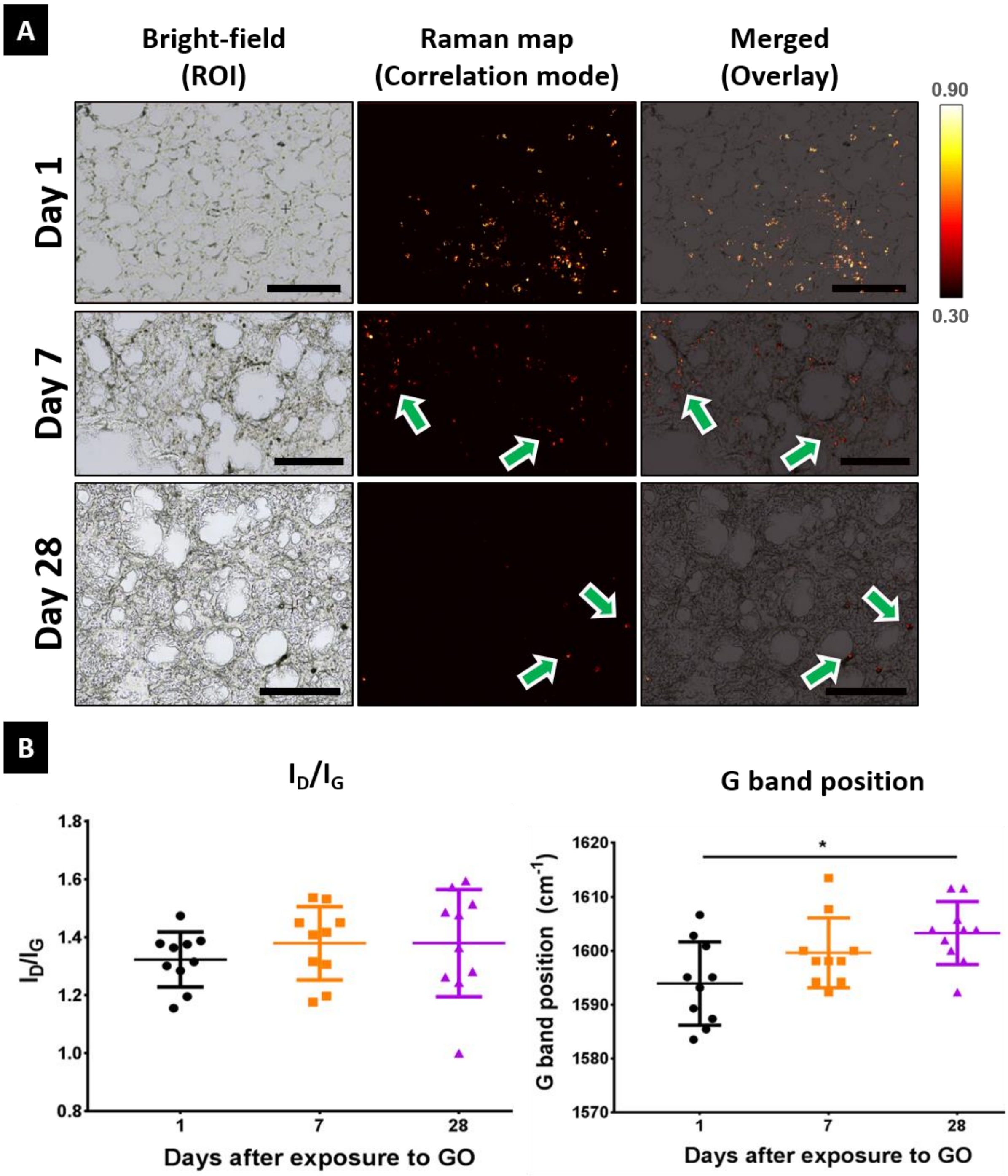

Figure S7. Raman mapping of lung sections from mice exposed to us-GO. Lungs were harvested from mice exposed to l-GO at days 1, 7 and 28 post exposure. **(A)** Raman spectroscopy revealed large areas of strong correlation indicating the presence of GO, as shown by the correlation maps overlaying the region of interest (ROI) in bright-field images. The decreased correlation at days 7 and 28 suggests the occurrence of clearance and/or material biotransformation. Scale bars = 200 µm. **(B)** Evolution of the structure of us-GO in

the lungs was assessed by calculating the ratio between the intensities of the D and G bands (I_D/I_G). Position of the G band was also evaluated to account for the emergence of the D' band. Individual data points correspond to 10 acquired spectra obtained from Raman maps, alongside mean values ± SD. Statistical analysis was performed using a Kruskal-Wallis test with *post hoc* Dunn's multiple comparisons test: **(*)**, $p < 0.05$.

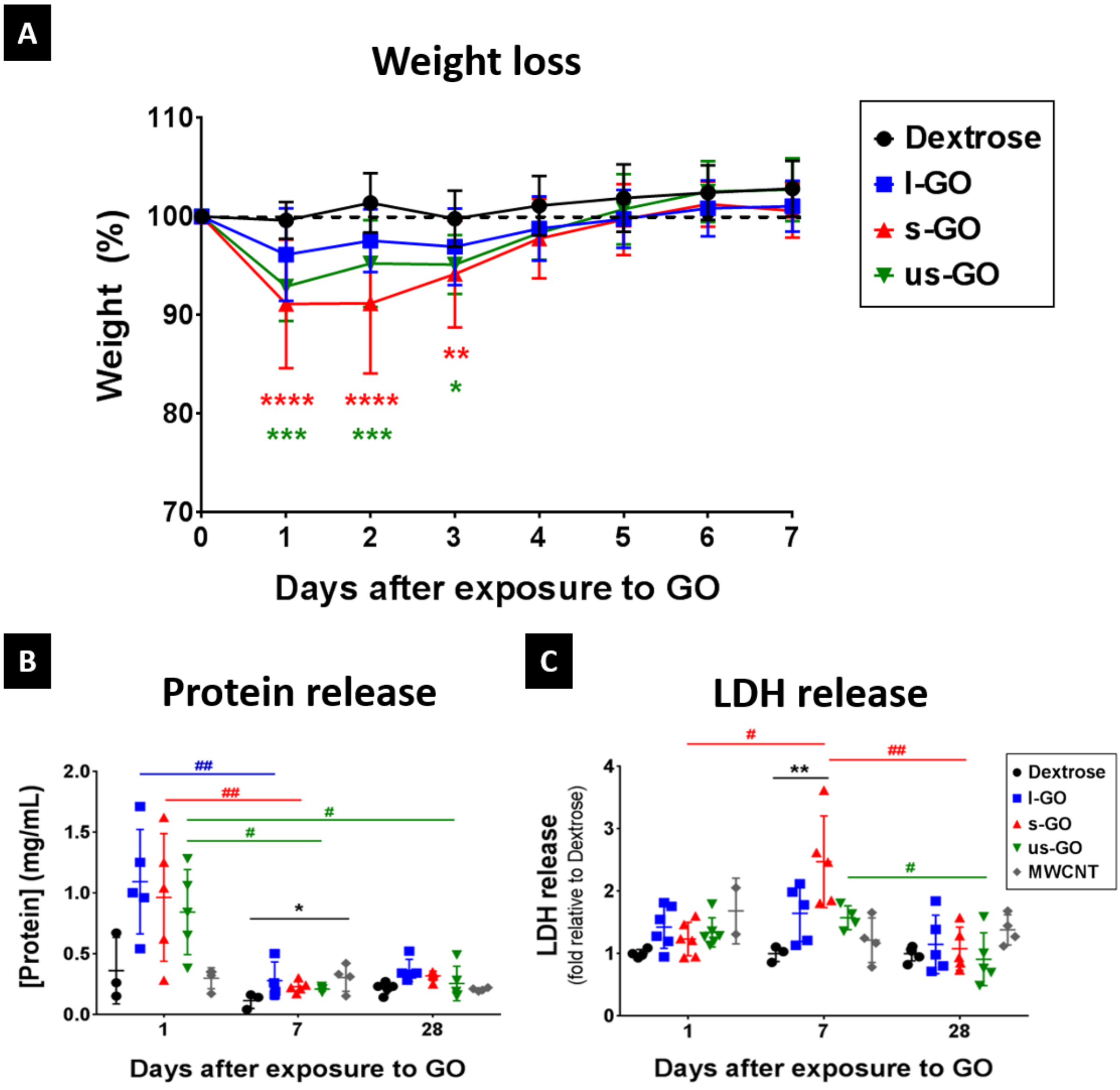

Figure S8. Acute pulmonary responses after intranasal instillation of GO. (A) Mice were frequently monitored up to 90 days. No significant differences in weight were observed among treatments after day 4 post exposure. Data are plotted as mean values ± SD and statistical analysis was performed using repeated-measures 2-way ANOVA test. Significant

differences compared to the vehicle-treated control (Dextrose) are reported as: **(*)**, $p < 0.05$; **(**)**, $p < 0.01$; **(***)**, $p < 0.001$; **(****)**, $p < 0.0001$. **(B, C)** The airways of mice exposed to GO or MWCNTs were lavaged with HBSS. After separation from the cell pellet by centrifugation, the supernatant of the BAL fluid was used to quantify the **(B)** total protein and **(C)** LDH release. Individual data points corresponding to each animal are plotted alongside mean values ± SD (n = 3-5). Statistical analysis was performed using a Kruskal-Wallis test with *post hoc* Dunn's multiple comparisons test against the negative control (Dextrose): **(*)**, $p < 0.05$; **(**)**, $p < 0.01$. The temporal variation of protein and LDH release in the airways was also analysed using a Kruskal-Wallis test with *post hoc* Dunn's multiple comparisons test: **(#)**, p < 0.05; **(##)**, $p < 0.01$.

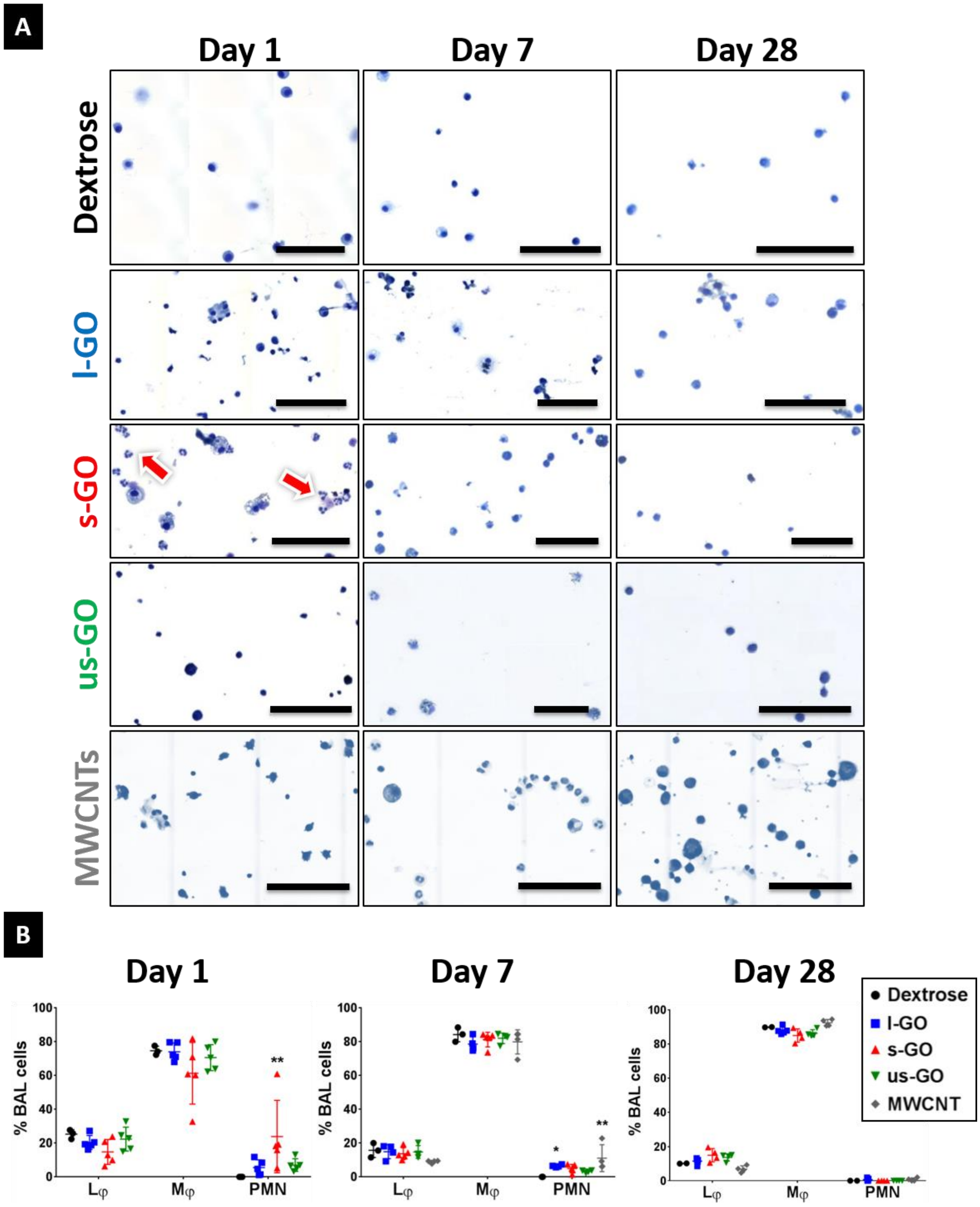

Figure S9. Recruitment of immune cells in the airways of mice exposed to carbon nanomaterials. Cells extracted from BAL fluid were differentially stained using Kwik-Diff™. **(A)** Representative images show an increased recruitment of neutrophils (indicated by arrows) to the airways of mice exposed to s-GO. Scale bars = 100 μm. **(B)** Recruitment of neutrophils and other polymorphonuclear (PMN) cells was lower for l-GO and us-GO,

despite an increased abundance of PMN in mice exposed to l-GO, 7 days after instillation. Relative abundance of lymphocytes (Lφ) and monocytic cells (Mφ) did not change significantly. Data in **(B)** are expressed as mean values ± SD (n = 4-5). One-way ANOVA was performed with Tukey's multiple comparisons test: **(*)**, $p < 0.05$; **(**)**, $p < 0.01$; **(***)**, $p < 0.001$; **(****)**, $p < 0.0001$.

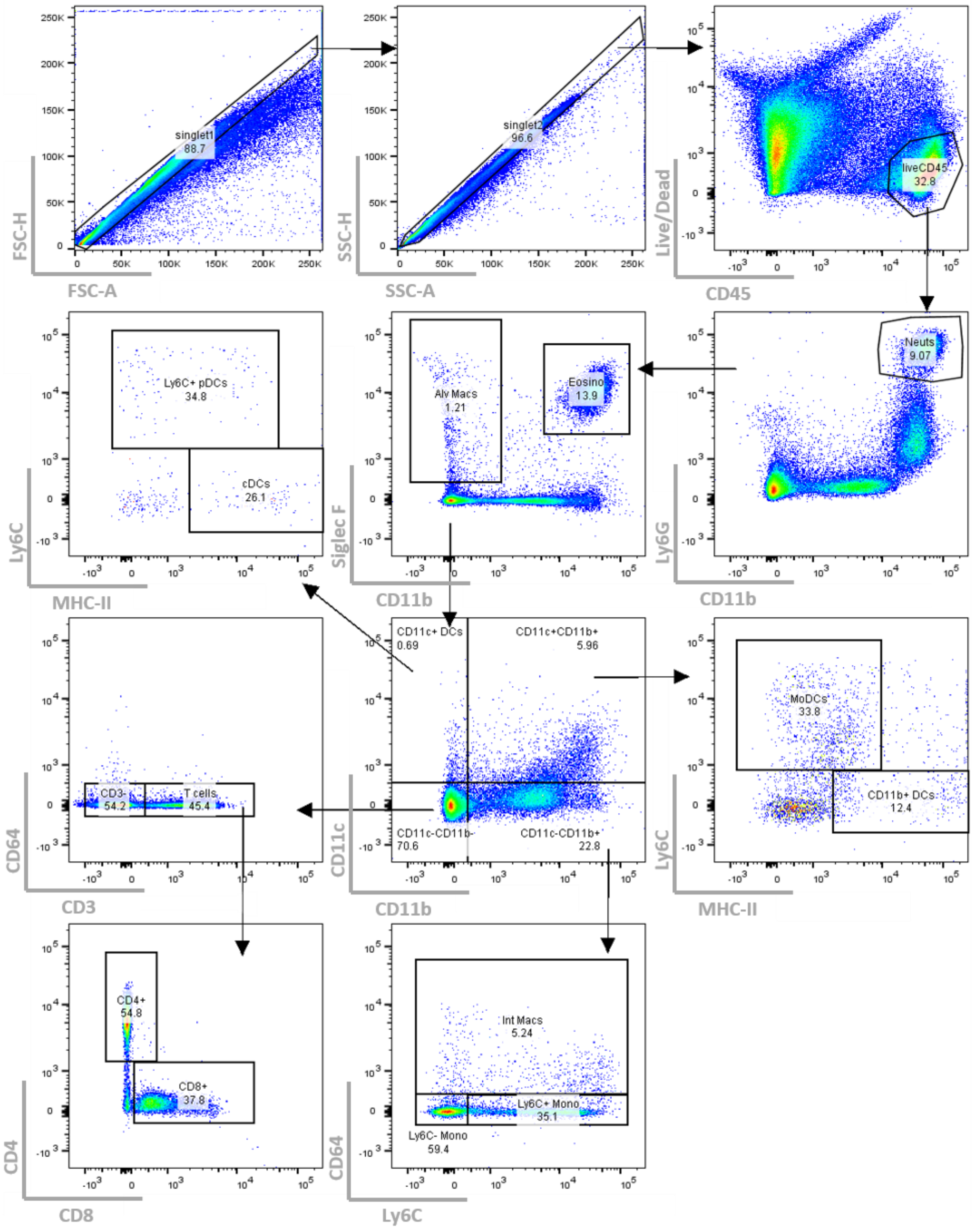

Figure S10. Gating strategy used in flow cytometry analysis to analyse immune cell subpopulations in digested lung tissue. Sample data from a control mouse is shown. Full description of gating strategy is presented in the **Methods** section.

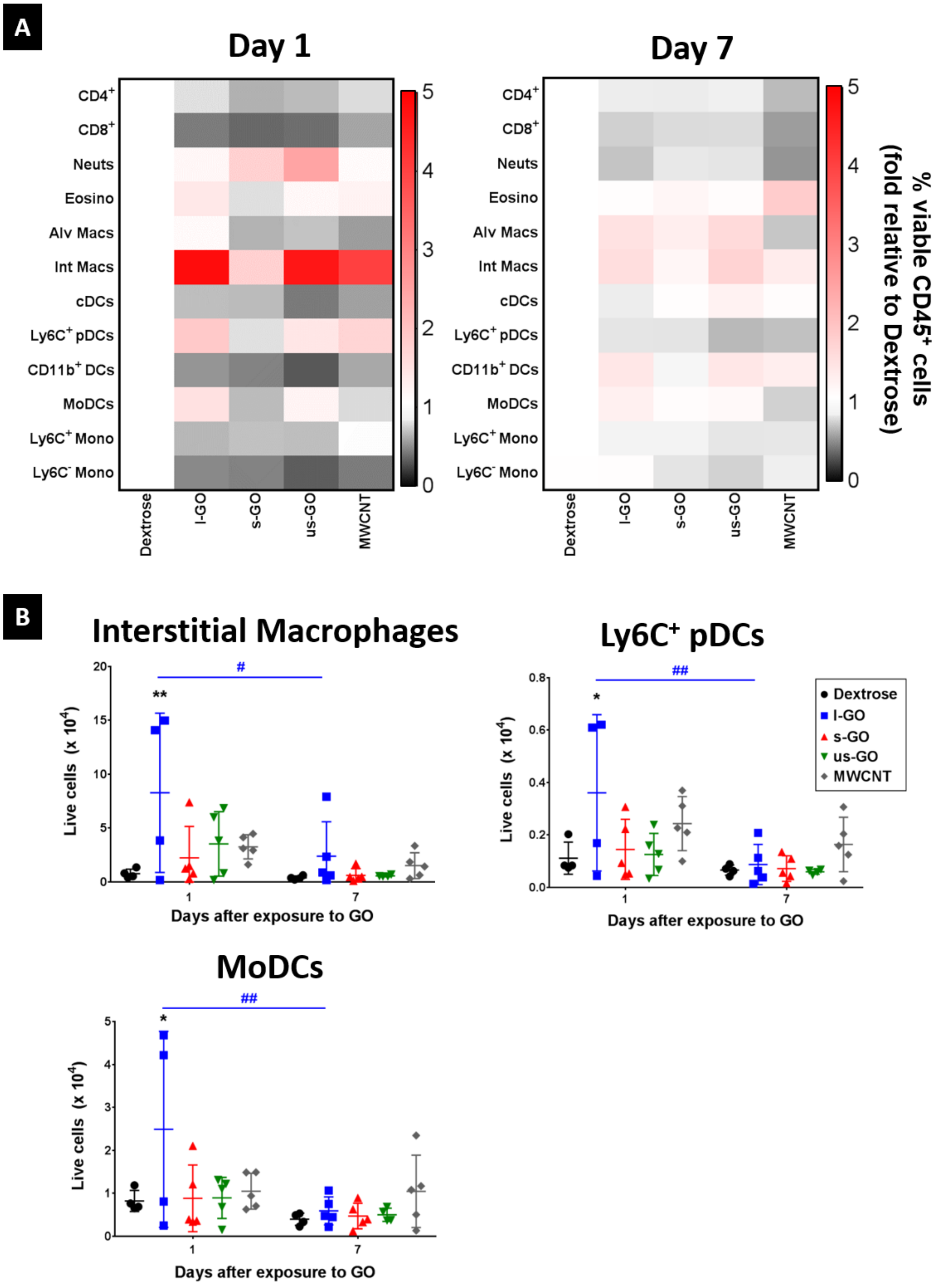

Figure S11. Relative quantification of immune cell populations in the lungs of mice exposed to GO. (A) Heatmap illustrates the variation in relative abundance as percentage of

live $CD45^+$ cells identified by flow cytometry, compared to the negative control (Dextrose). Each block represents the mean value, after normalisation to the average total number of cells in a specific immune cell population (n = 4). **(B)** Major immune cell subpopulations that revealed statistically significant differences compared to the negative control. Individual data points corresponding to each animal are plotted alongside mean values ± SD (n = 4). Statistical analysis was performed using a 2-way ANOVA test with *post hoc* Sidak's multiple comparisons test compared to the Dextrose control. Significant differences between treatments are plotted with (*), whereas differences over time are plotted with (#). In both cases, statistical significance is reported as: **(*)**, $p < 0.05$; **(**)**, $p < 0.01$.

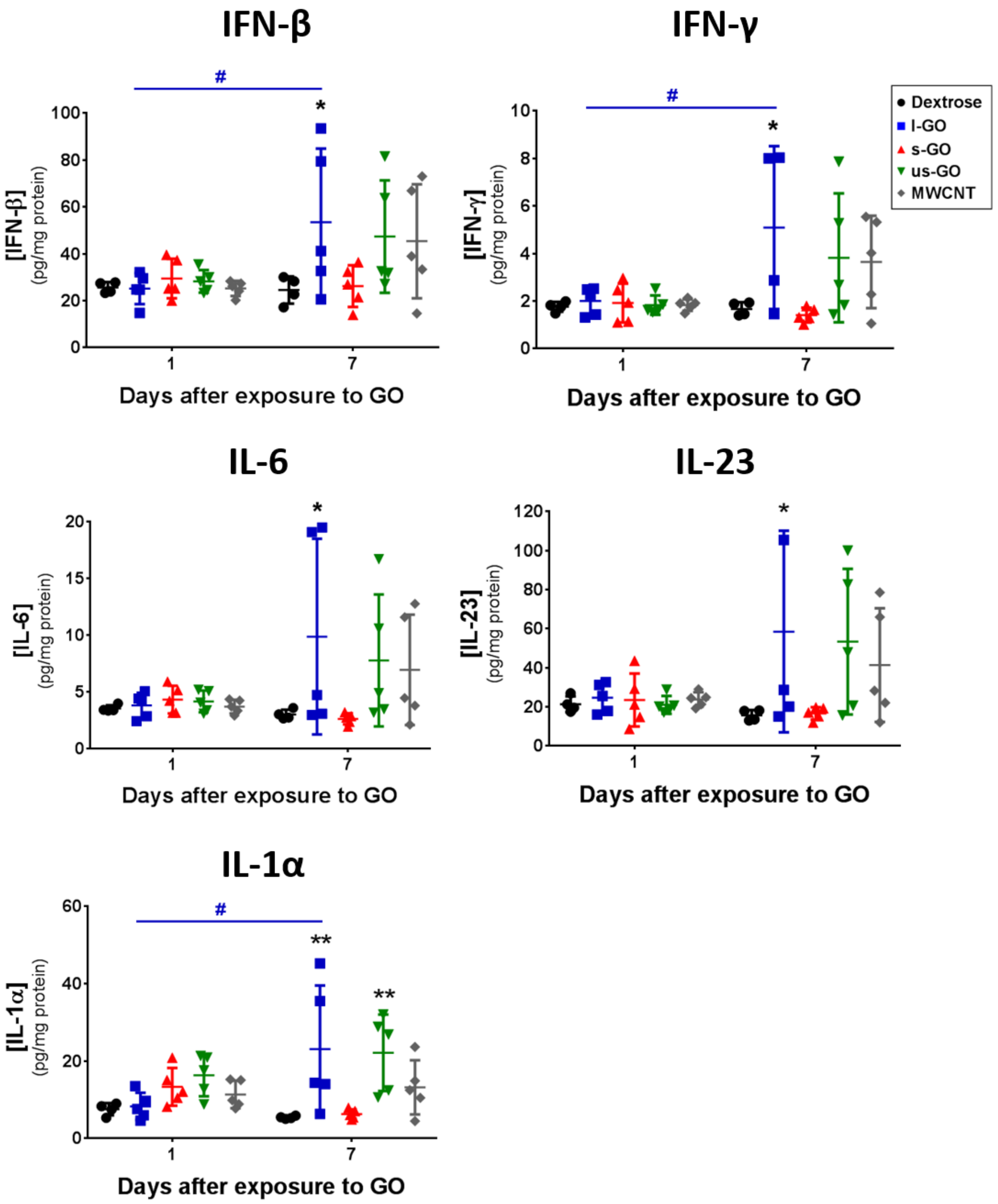

Figure S12. Upregulation of relevant pro-inflammatory cytokines in the lungs after exposure to l-GO. Small portions of each individual lobe were dissected for protein extraction. Cytokine secretion was quantified using a LEGENDplex Mouse Inflammation Panel kit, and normalised to protein concentration determined by BCA assay. Individual data

points corresponding to each animal are plotted alongside mean values ± SD (n = 4-5). Statistical analysis was performed using a 2-way ANOVA test with *post hoc* Sidak's multiple comparisons test compared to the Dextrose control. Significant differences between treatments are plotted with (*), whereas differences over time are plotted with (#). In both cases, statistical significance is reported as: **(*)**, $p < 0.05$.

A

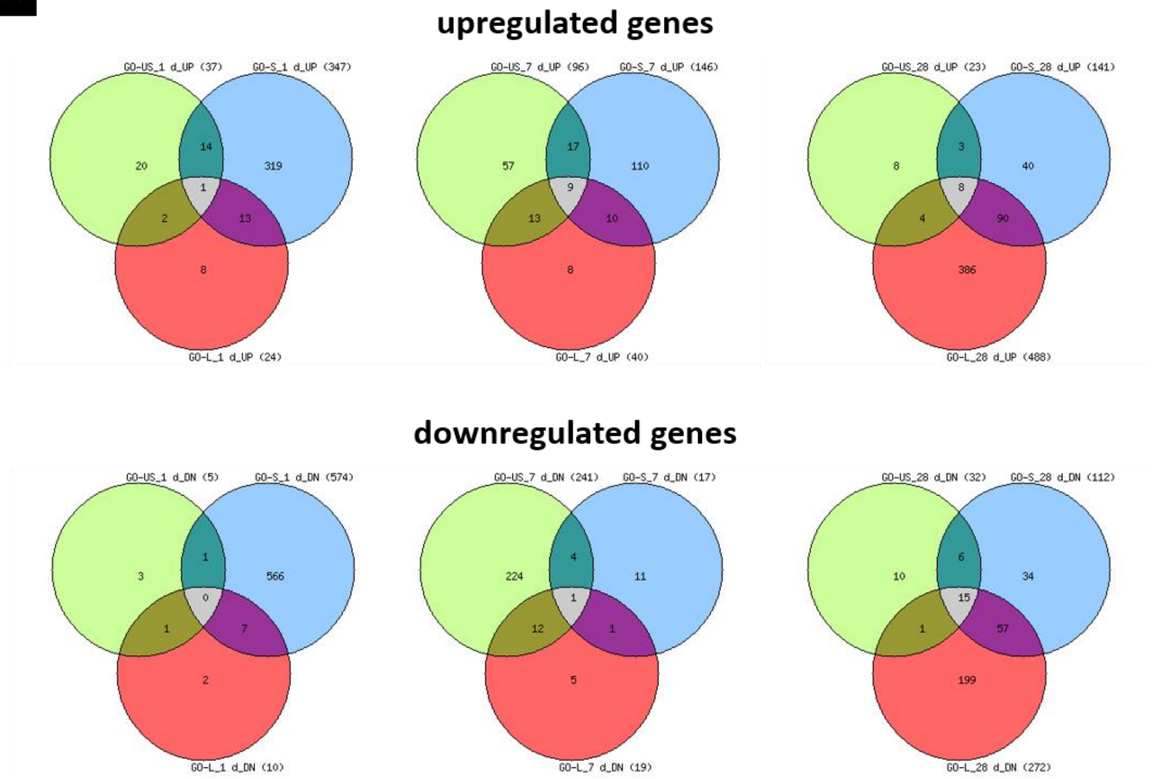

upregulated genes
GO-US_1 d_UP (37)
GO-S_1 d_UP (347)
GO-L_1 d_UP (24)
20
14
319
1
2
13
8
GO-US_7 d_UP (96)
GO-S_7 d_UP (146)
GO-L_7 d_UP (40)
57
17
110
9
13
10
8
GO-US_28 d_UP (23)
GO-S_28 d_UP (141)
GO-L_28 d_UP (488)
8
3
40
8
4
90
386
downregulated genes
GO-US_1 d_DN (5)
GO-S_1 d_DN (574)
GO-L_1 d_DN (10)
3
1
566
0
1
7
2
GO-US_7 d_DN (241)
GO-S_7 d_DN (17)
GO-L_7 d_DN (19)
224
4
11
1
12
1
5
GO-US_28 d_DN (32)
GO-S_28 d_DN (112)
GO-L_28 d_DN (272)
10
6
34
15
1
57
199

B

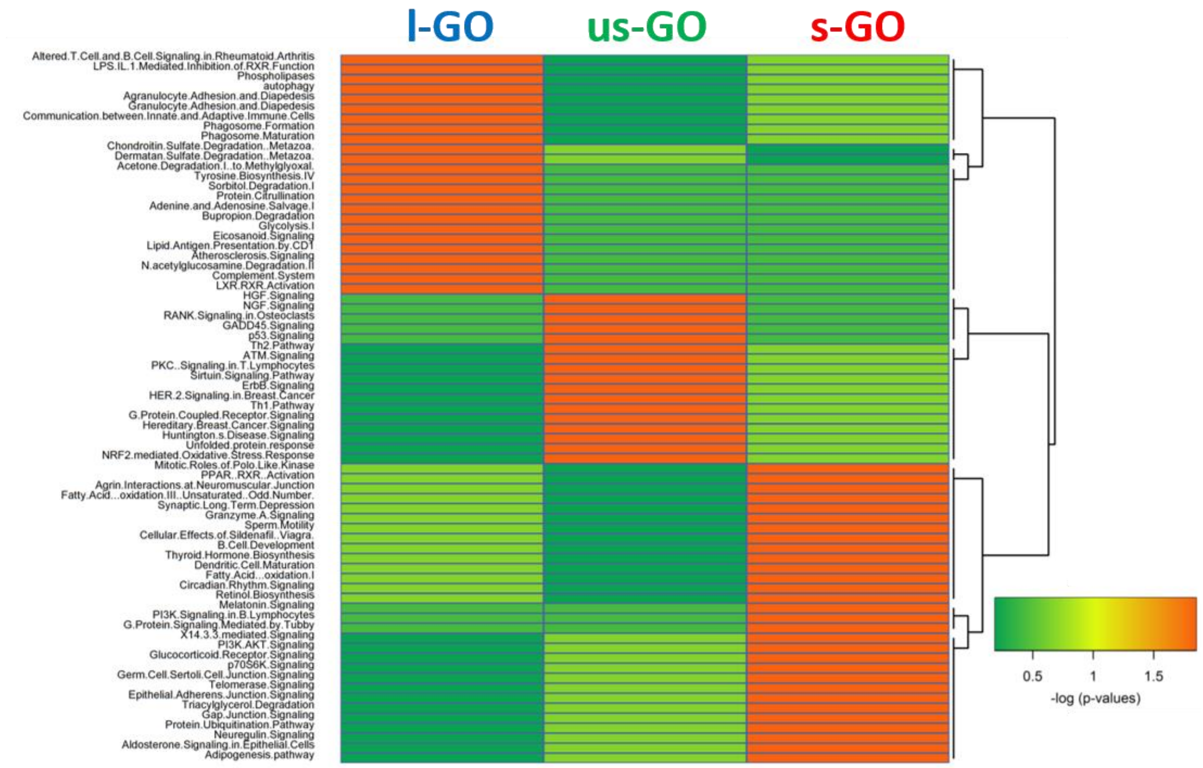

l-GO
us-GO
s-GO
Altered.T.Cell.and.B.Cell.Signaling.in.Rheumatoid.Arthritis
LPS.IL.1.Mediated.Inhibition.of.RXR.Function
Phospholipases
autophagy
Agranulocyte.Adhesion.and.Diapedesis
Granulocyte.Adhesion.and.Diapedesis
Communication.between.Innate.and.Adaptive.Immune.Cells
Phagosome.Formation
Phagosome.Maturation
Chondroitin.Sulfate.Degradation..Metazoa.
Dermatan.Sulfate.Degradation..Metazoa.
Acetone.Degradation.I..to.Methylglyoxal.
Tyrosine.Biosynthesis.IV
Sorbitol.Degradation.I
Protein.Citrullination
Adenine.and.Adenosine.Salvage.I
Bupropion.Degradation
Glycolysis.I
Eicosanoid.Signaling
Lipid.Antigen.Presentation.by.CD1
Atherosclerosis.Signaling
N.acetylglucosamine.Degradation.II
Complement.System
LXR.RXR.Activation
HGF.Signaling
NGF.Signaling
RANK.Signaling.in.Osteoclasts
GADD45.Signaling
p53.Signaling
Th2.Pathway
ATM.Signaling
PKC..Signaling.in.T.Lymphocytes
Sirtuin.Signaling.Pathway
ErbB.Signaling
HER.2.Signaling.in.Breast.Cancer
Th1.Pathway
G.Protein.Coupled.Receptor.Signaling
Hereditary.Breast.Cancer.Signaling
Huntington.s.Disease.Signaling
Unfolded.protein.response
NRF2.mediated.Oxidative.Stress.Response
Mitotic.Roles.of.Polo.Like.Kinase
PPAR..RXR..Activation
Agrin.Interactions.at.Neuromuscular.Junction
Fatty.Acid...oxidation.III..Unsaturated..Odd.Number.
Synaptic.Long.Term.Depression
Granzyme.A.Signaling
Sperm.Motility
Cellular.Effects.of.Sildenafil..Viagra.
B.Cell.Development
Thyroid.Hormone.Biosynthesis
Dendritic.Cell.Maturation
Fatty.Acid...oxidation.I
Circadian.Rhythm.Signaling
Retinol.Biosynthesis
Melatonin.Signaling
PI3K.Signaling.in.B.Lymphocytes
G.Protein.Signaling.Mediated.by.Tubby
X14.3.3.mediated.Signaling
PI3K.AKT.Signaling
Glucocorticoid.Receptor.Signaling
p70S6K.Signaling
Germ.Cell.Sertoli.Cell.Junction.Signaling
Telomerase.Signaling
Epithelial.Adherens.Junction.Signaling
Triacylglycerol.Degradation
Gap.Junction.Signaling
Protein.Ubiquitination.Pathway
Neuregulin.Signaling
Aldosterone.Signaling.in.Epithelial.Cells
Adipogenesis.pathway
0.5
1
1.5
-log (p-values)

Figure S13. RNA-seq reveals time-dependent changes in gene expression following exposure to GO. **(A)** Venn diagrams of differentially expressed genes from transcriptomics analysis of lung tissue samples of C57BL/6 mice intranasally exposed to us-GO, s-GO, and l-GO for 1, 7 and 28 days. Upregulated genes are depicted on top, whereas downregulated genes are presented in the bottom. DEGs having ≥ 0.5 log fold change and ≥ 0.05 false discovery rate were included in the analysis. **(B)** Hierarchical clustering analysis of the top *canonical pathways* identified by IPA in lung tissue samples of mice exposed for 28 days to us-GO, s-GO, and l-GO. DEGs having ≥ 0.5 log fold change and ≥ 0.05 false discovery rate were included in the analysis.

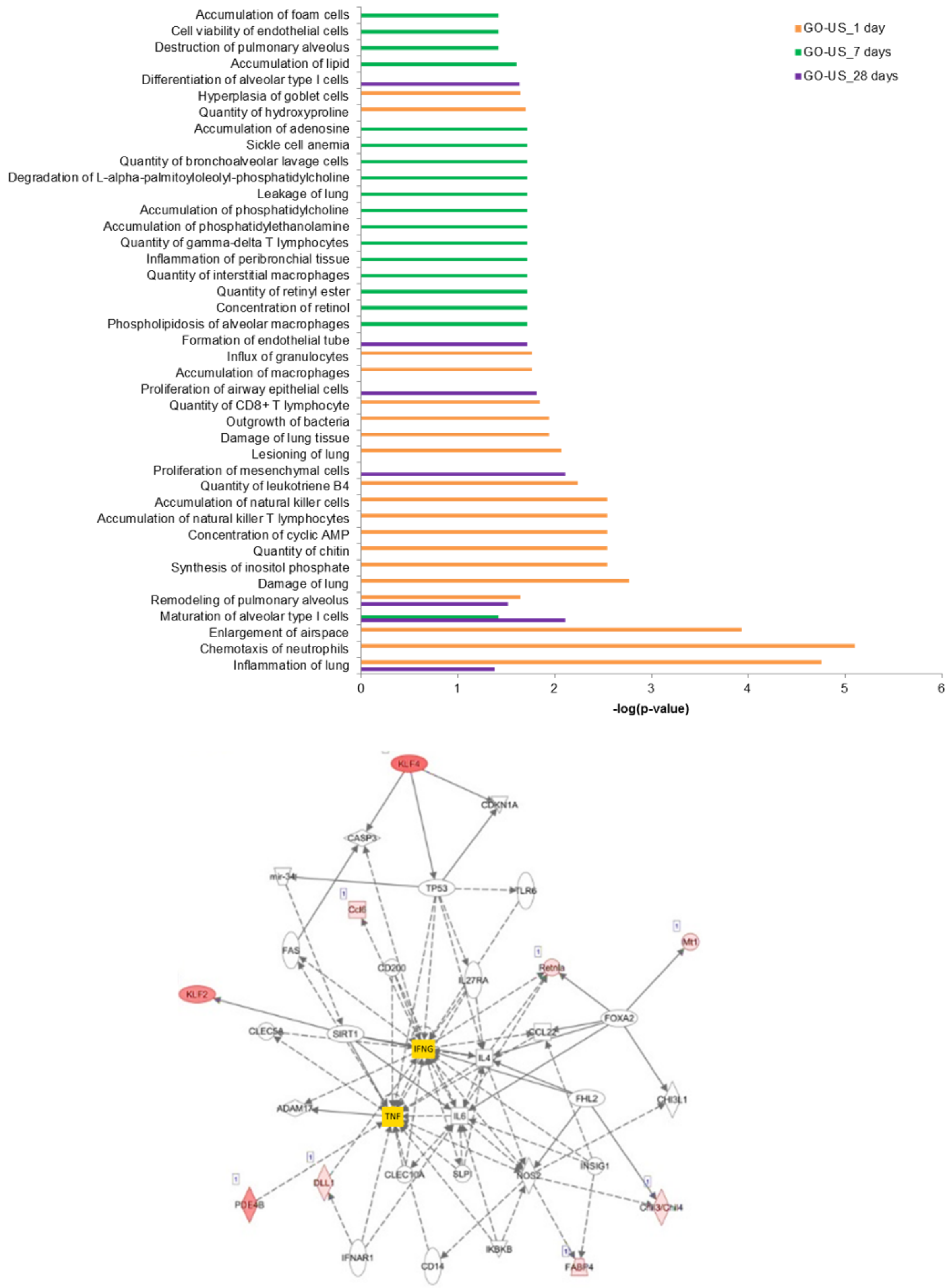

Figure S14. Significantly affected gene expression pathways upon exposure to us-GO.

The top *diseases and biofunctions* pathways identified by using the IPA software in lung tissue samples of mice exposed for 1, 7, and 28 days to a single dose of us-GO. DEGs having

≥ 0.5 log fold change and ≥ 0.05 FDR were included in the analysis. Network analysis of DEGs identified in the lungs of mice at 28 days post-exposure was generated by IPA to predict which activators affected gene expression.

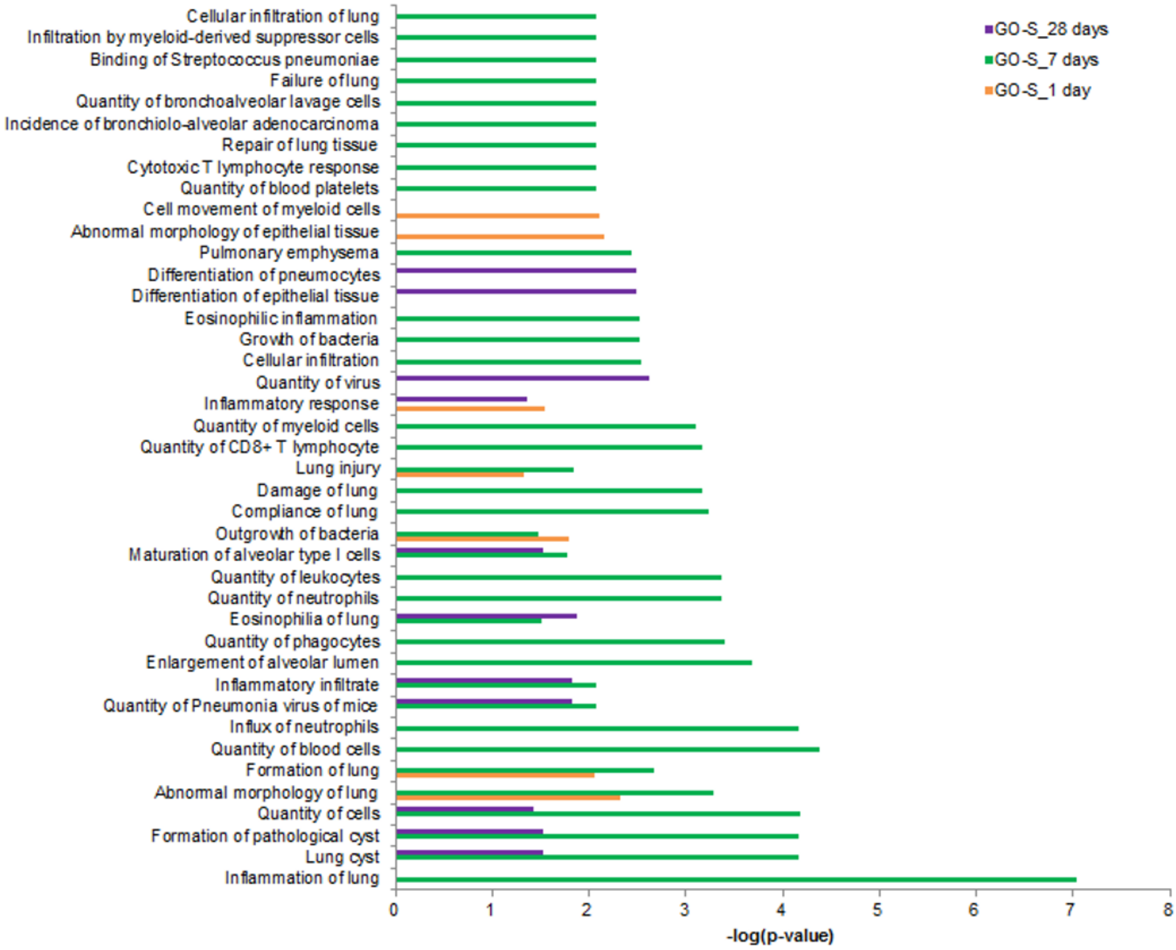
Cellular infiltration of lung
Infiltration by myeloid-derived suppressor cells
Binding of Streptococcus pneumoniae
Failure of lung
Quantity of bronchoalveolar lavage cells
Incidence of bronchiolo-alveolar adenocarcinoma
Repair of lung tissue
Cytotoxic T lymphocyte response
Quantity of blood platelets
Cell movement of myeloid cells
Abnormal morphology of epithelial tissue
Pulmonary emphysema
Differentiation of pneumocytes
Differentiation of epithelial tissue
Eosinophilic inflammation
Growth of bacteria
Cellular infiltration
Quantity of virus
Inflammatory response
Quantity of myeloid cells
Quantity of CD8+ T lymphocyte
Lung injury
Damage of lung
Compliance of lung
Outgrowth of bacteria
Maturation of alveolar type I cells
Quantity of leukocytes
Quantity of neutrophils
Eosinophilia of lung
Quantity of phagocytes
Enlargement of alveolar lumen
Inflammatory infiltrate
Quantity of Pneumonia virus of mice
Influx of neutrophils
Quantity of blood cells
Formation of lung
Abnormal morphology of lung
Quantity of cells
Formation of pathological cyst
Lung cyst
Inflammation of lung
0
1
2
3
4
5
6
7
8
-log(p-value)
GO-S_28 days
GO-S_7 days
GO-S_1 day

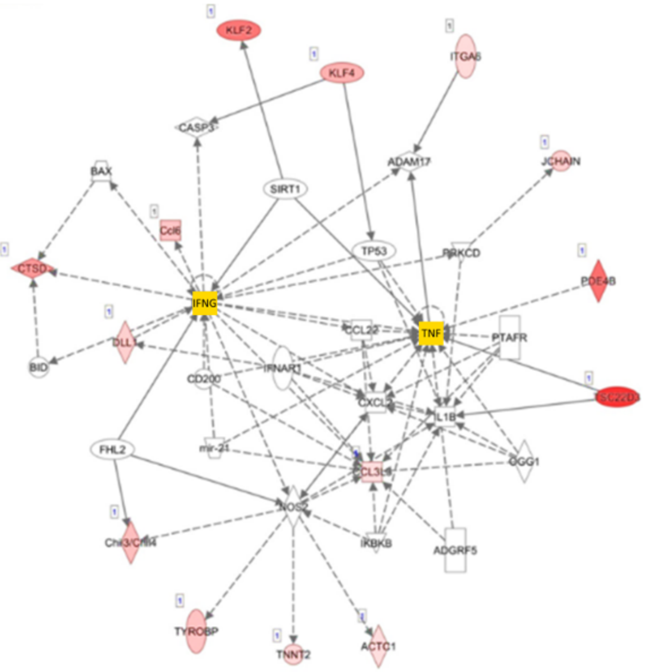
KLF2
KLF4
ITGA6
CASP3
BAX
ADAM17
JCHAIN
SIRT1
Ccl6
TP53
PRKCD
CTSD
PDE4B
IFNG
CCL22
TNF
PTAFR
DLL1
BID
IFNAR1
CD200
CXCL2
IL1B
FHL2
mir-21
OGG1
Chi3/Chil4
IKBKB
ADGRF5
TYROBP
TNNT2
ACTC1

Figure S15. Significantly affected gene expression pathways upon exposure to s-GO. The top *diseases and biofunctions* pathways identified by using the IPA software in lung tissue samples of mice exposed for 1, 7, and 28 days to a single dose of s-GO. DEGs having ≥ 0.5 log fold change and ≥ 0.05 FDR were included in the analysis. Network analysis of DEGs identified in the lungs of mice at 28 days post-exposure was generated by IPA to predict which activators affected gene expression.

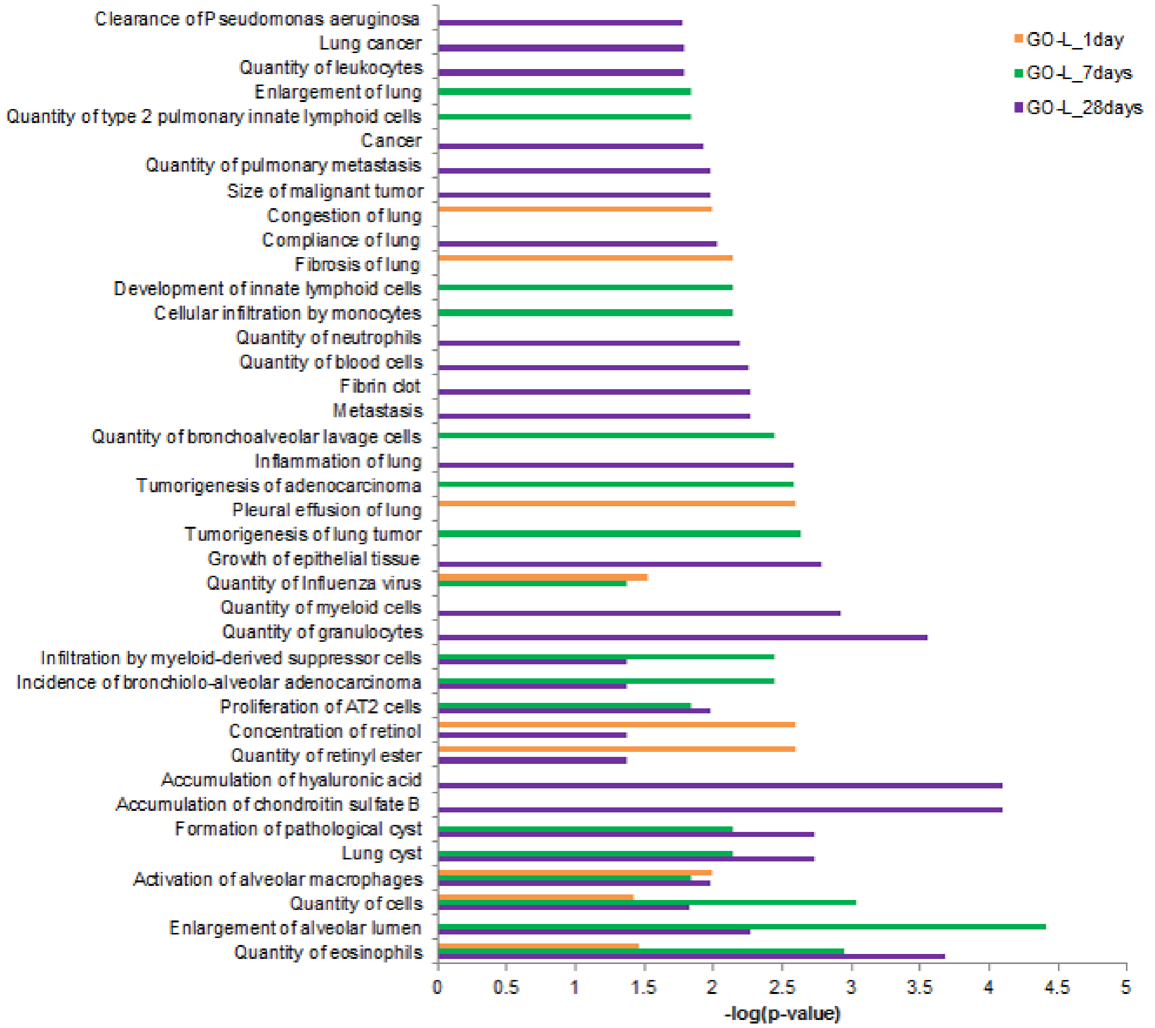

Figure S16. Significantly affected gene expression pathways upon exposure to l-GO. The top *diseases and biofunctions* pathways identified by using the IPA software in lung tissue samples of mice exposed for 1, 7, and 28 days to a single dose of l-GO. DEGs having ≥ 0.5 log fold change and ≥ 0.05 FDR were included in the analysis.

SUPPLEMETARY CALCULATIONS

Extrapolation of instilled dose in mice to human exposure. The human equivalent exposure dose (D_{human}) of GO was estimated based on the deposition fractions obtained in the lungs of mice after i.n. instillation [70]:

$$\frac{50\ \mu g}{0.05\ m^2} \times F = \frac{D_{human}}{102.2\ m^2} \times 30\% \quad \textbf{(1)}$$

where F represents the deposited fraction of GO in the lower respiratory tract, which is the total relative instilled dose detected in lungs and trachea (see **Table S1, Supporting Information**). Human deposition efficiency of inhaled particles was estimated to be about 30% [24]. Interspecies extrapolation considered the normalisation of lung burden by their respective airway surface area, which is 102.2 m^2 for humans and 0.05 m^2 for mice [71].

We therefore calculated the human equivalent exposure doses for the 3 GO materials:

- l-GO: $F = 7.01\% \pm 4.42\% \Rightarrow D_{human} = 23.87 \pm 15.05\ mg$
- s-GO: $F = 17.48\% \pm 7.44\% \Rightarrow D_{human} = 59.55 \pm 25.33\ mg$
- us-GO: $F = 12.08\% \pm 5.77\% \Rightarrow D_{human} = 41.16 \pm 19.65\ mg$

Based on aerosol inhalation studies, the expected daily exposed dose in humans is calculated as:

$$Exposed\ dose\ (mg/day) = C_{ae}\ (mg/m^3) \times 0.02\ m^3/min \times 60\ min/h \times\ 8\ h/day \quad \textbf{(2)}$$

where C_{ae} is the concentration of the generated aerosol, and an average breathing rate of 20 L/min is assumed [24].

We therefore divided the equivalent human exposure doses, determined from **Equation 1**, by the estimated daily exposed dose by inhalation obtained in **Equation 2**, in order to estimate the number of working days needed to have an equivalent lung burden. We fixed C_{ae} to relevant occupational exposure settings, between the recommended limit of 1 μg/m^3 and detected levels of 50 μg/m^3 of carbon nanomaterials at manufacturing sites [72]:

**Material**	C_{ae} **(μg/m^3)**		
	**1**	**10**	**50**
**l-GO**	2486.4 ± 1568.0 days	248.6 ± 156.8 days	49.7 ± 31.4 days
**s-GO**	6203.6 ± 2639.0 days	620.4 ± 263.9 days	124.1 ± 52.8 days
**us-GO**	4287.5 ± 2047.2 days	428.8 ± 204.7 days	85.8 ± 40.9 days

Assuming that 1 month has 22 working days and 1 year has 264 working days (22 working days × 12 months), the values in the table above were converted to years of occupational exposure:

**Material**	C_{ae} **(μg/m^3)**	
	**1**	**10**
**l-GO**	9.4 ± 5.9 years	0.9 ± 0.6 years
**s-GO**	23.5 ± 10.0 years	2.3 ± 1.0 years
**us-GO**	16.2 ± 7.8 years	1.6 ± 0.8 years

At the highest exposure concentration of 50 μg/m^3, the estimated exposure times are:

- l-GO: 2.3 ± 1.4 months

- s-GO: 5.6 ± 2.4 months
- us-GO: 3.9 ± 1.9 months

Extrapolation of inhaled dose in rats to deposited dose in mice. Considering that Kim *et al*. reported a total deposited dose of 0.515 mg over 5 days and estimated a deposition efficiency of 15.36% [36], **Equation 1** was replaced to estimate the mouse equivalent exposure dose (D_{mouse}), after adjusting the surface area to 0.4 m^2, which corresponds to the rat pulmonary epithelium [71]:

$$\frac{D_{mouse}}{0.05\ m^2} \times F = 0.515\ mg \Rightarrow D_{mouse} = 0.515\ mg \times \frac{1}{F} \times \frac{0.05\ m^2}{0.4\ m^2}$$

Assuming $F = 20\%$ as observed after i.n. instillation of s-GO, the equivalent exposure dose would be $D_{mouse} = 322\ \mu g$, which is equivalent to 5 daily exposures of 64.4 µg
